# High-performance electrochemical biosensor comprising Mn-ZIF-67 conjugated with anti-O antibody for Escherichia coli detection

**DOI:** 10.1038/s42004-025-01703-y

**Published:** 2025-10-01

**Authors:** Atqiya Muslihati, Chandra Wulandari, Ni Luh Wulan Septiani, Gilang Gumilar, Agus Subagio, Ida Hamidah, Nugraha Nugraha, Erwin Peiner, Hutomo Suryo Wasisto, Brian Yuliarto

**Affiliations:** 1 Doctoral Program of Engineering Physics, Faculty of Industrial Technology, Institut Teknologi Bandung, Bandung, Indonesia; 2 Advanced Functional Material Laboratory, Faculty of Industrial Technology, Institut Teknologi Bandung, Bandung, Indonesia; 3 PT Biostark Analitika Inovasi, Bandung, Indonesia; 4 Research Center for Electronics, National Research and Innovation Agency (BRIN), Bandung, Indonesia; 5 Department of Physics, Faculty of Science and Mathematics, Universitas Diponegoro, Semarang, Indonesia; 6 Department of Engineering and Vocational Education, Universitas Pendidikan Indonesia, Bandung, Indonesia; 7 Research Center for Nanosciences and Nanotechnology (RCNN), Institut Teknologi Bandung, Bandung, Indonesia; 8 Institute of Semiconductor Technology (IHT) and Laboratory for Emerging Nanometrology (LENA), Technische Universität Braunschweig, Braunschweig, Germany

**Keywords:** Biophysical chemistry, Sensors and biosensors, Design, synthesis and processing, Electrochemistry

## Abstract

The detection of *Escherichia coli* (*E. coli*) in food and water is critical for public health, yet existing methods often lack sensitivity, speed, or field applicability. Here, we develop an electrochemical biosensor based on Mn-doped Co zeolitic imidazolate framework (ZIF-67) functionalized with anti-O antibody for sensitive *E. coli* detection. Mn incorporation induces phase reconstruction, surface area enhancement, and electron transfer. Antibody conjugation modulates wettability, introduces amide I and II vibrational modes, and selectively blocks electron transfer upon bacterial binding. The biosensor exhibits a linear range of 10 to 10^10^ CFU mL^–1^ with a 1 CFU mL^–1^ detection limit, outperforming optical and other metal organic framework-based sensors. It can discriminate non-target bacteria (*Salmonella, Pseudomonas aeruginosa, Staphylococcus aureus*), maintain >80% sensitivity over 5 weeks, and recover 93.10 –107.52% *E. coli* spiked in tap water. This work suggests the tremendous potential for on-site pathogen monitoring.

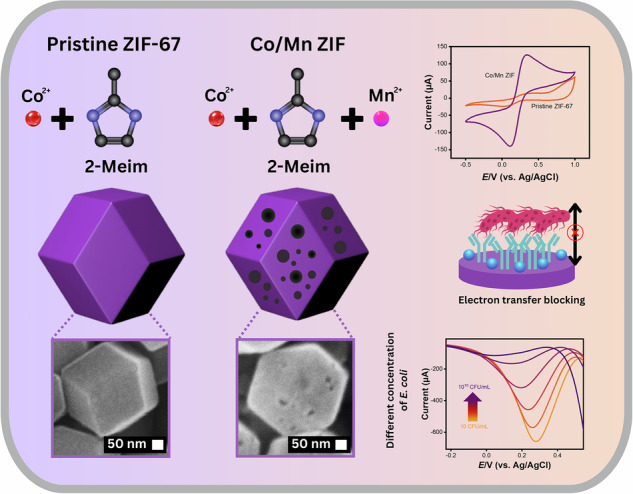

## Abstract (web summary)

Zeolitic imidazolate (ZIF-67), a Co-based metal organic framework, is a promising candidate for *Escherichia coli* detection, but its application is hampered by poor electrical conductivity. Here, the authors present a Co/Mn-based ZIF-67 that leverages the synergistic action of two transition metals to enhance sensor sensitivity, demonstrating its potential for on-site pathogen monitoring.

## Introduction

E*scherichia coli* (*E. coli*) is a significant microorganism widely used as a marker for evaluating food and water safety^1,2^. Pathogenic *E. coli* resides in various animal reservoirs and can be transmitted through fecal contamination in food, water, or irrigation systems^3^. Several diseases are reported due to pathogenic *E. coli* contamination, which causes diarrhea, urinary tract infections, bacteremia, and cholecystitis^4–6^. However, conventional culture methods are time-consuming, requiring 2–10 days for confirmation^7^. Biosensors have undergone great strides, allowing rapid and accurate bacterial detection through interactions between target analytes and bioreceptors, which occur on the electrode material surface that is linked to a specific transducer, resulting in a biochemical reaction into the data readout^8–10^. Functionalization is key in biosensor surface modification, facilitating sensitivity and selectivity between the target analytes and bioreceptors, and ensuring signal transmission to the transducer^11–15^. This functionalization involves several level modifications (i.e., physicochemical surface activation and bioreceptor immobilization). Optimizing the physicochemical and electrical properties of the surface layer boosts the transducer sensitivity, while modifying the bioreceptor may enhance selectivity by further refining the surface layer^16–18^.

Zeolitic imidazolate framework (ZIF-67), a Co-based metal-organic framework (MOF) with 2-methylimidazole as the ligand, exhibits excellent crystallinity and high chemical stability^19,20^. Its large surface area allows modifications (e.g., conjugation and immobilization)^21^, while its porous structure promotes electron transfer during redox reactions, thereby improving physicochemical properties^22^. Despite these promising properties, ZIF-67 has poor intrinsic electrical conductivity^23^. Recent studies focus on metal doping and structural modifications to enhance its conductivity and surface reactivity^24–26^. By integrating a secondary metal ion in the framework, bimetallic centers in MOFs obtain synergistic effects^27,28^. Catalytic effectiveness is enhanced by the incorporation of Ni into Co ZIF-67^29^. For instance, adding manganese to Fe-based MOFs offers them enzyme-like catalytic activity against hydroquinones^30^, whereas adding Mn-Co MOFs produces higher conductivity than Mn MOFs themselves^31^. Mn, in particular, accelerates catalytic performance and supports applications, e.g., cancer labeling, bacterial impedance amplification, and glucose detection^32–36^. The favorable size of Mn ions and their optimal electron orbital configuration maintain the MOF’s structural integrity through the incorporation of Mn into the MOF host^37^.

Previous bimetallic MOF-based biosensors targeting *E. coli* have typically exhibited higher detection limits depending on the used transducer and bioreceptor, which relied on broad-spectrum anti-*E. coli* antibodies^38–46^. Our earlier study disclosed that incorporating Mn outstandingly boosts electron transfer as supported by electrochemical cyclic voltammetry (CV) measurements^47^. Building upon this, we present a Co/Mn-based ZIF-67 (Co/Mn ZIF) composite, leveraging the synergistic action of two transition metals to enhance sensor sensitivity. This study systematically investigates how varying Mn content affects electron transfer and key structural features, including crystallinity, surface area, porosity, and morphology, to determine the optimal configuration for sensing applications. To date, no prior study has reported the development of a bimetallic ZIF-based MOF specifically tailored for the detection of *E. coli*. Although the modifications of MOFs have been broadly investigated, the study of Mn – especially within the Co ZIF-67 framework for sensing purposes – has been partially overlooked. This study conjugates anti-O-specific antibodies that bind selectively to the O-polysaccharide region of *E. coli*, thereby optimizing the selectivity of the biosensor. By leveraging the inherent advantages of electrochemical biosensors (e.g., high sensitivity, stability, and cost-effectiveness)^48–50^, we introduce a sensing platform that integrates bimetallic MOF with selective bioreceptors. The goal is to develop a highly sensitive and selective electrochemical biosensor for *E. coli* detection, achieving a low detection limit while maintaining reliable performance in real sample testing.

## Results and discussion

### Physicochemical properties of Co/Mn ZIF materials

**Fig. 1:**
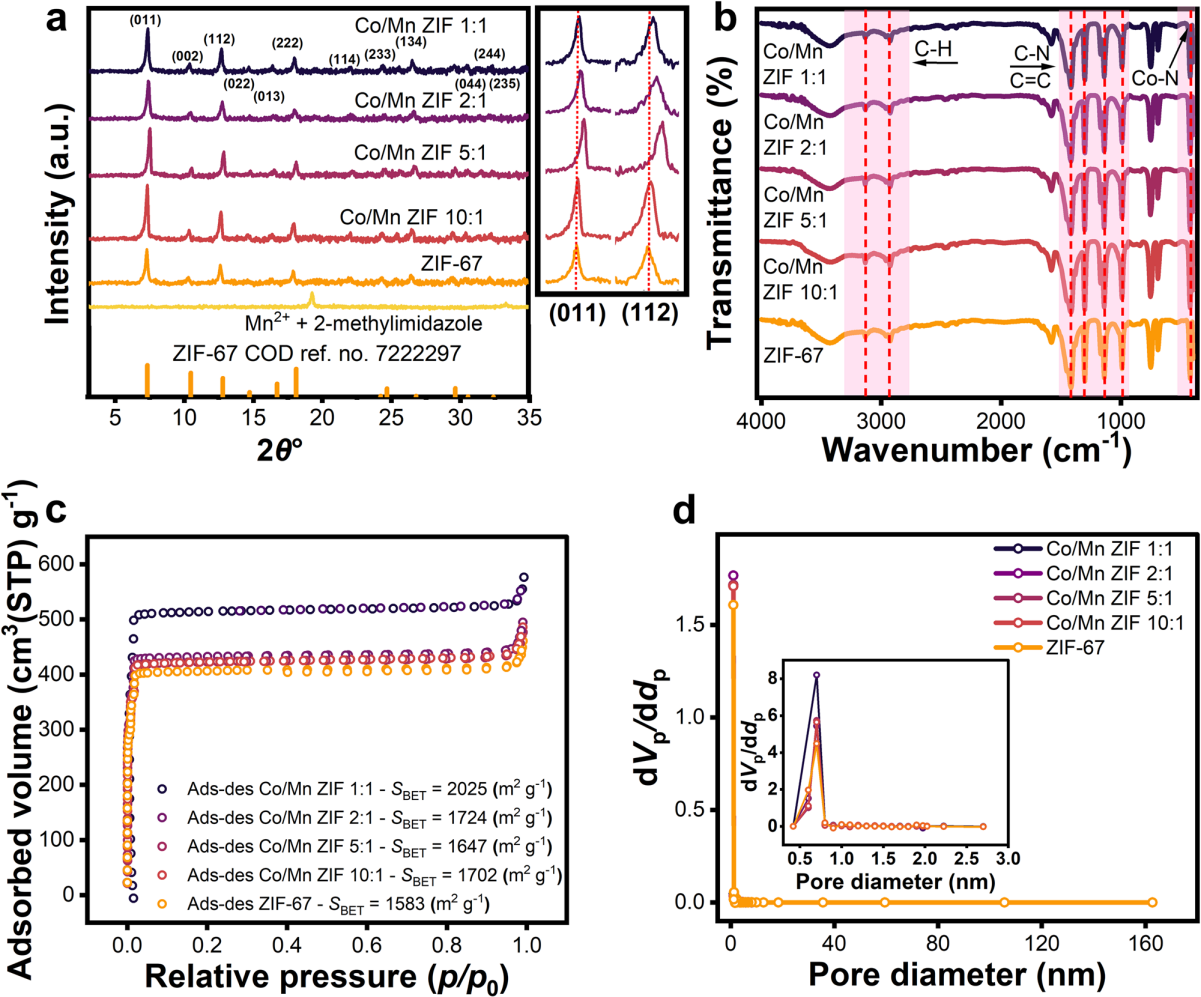
Structural characterizations of Co/Mn ZIF at different ratios of Co^2+^ and Mn^2+^. **a** X-ray diffraction (XRD) patterns of Co/Mn ZIF compared to a reference pattern (COD 7222297), showing the incorporation of Mn^2+^ into ZIF-67 host via peak shifts in the (011) and (112) planes (right side box). **b** Fourier-transform infrared (FTIR) spectra of Co/Mn ZIF and ZIF-67 confirm functional group preservation with no Mn^2+^-induced peak changes. **c** Type I N_2_ adsorption–desorption isotherm of Co/Mn ZIF, outlining the largest surface area (dark-blue circle) based on the Brunauer–Emmett–Teller (BET) method with a large N_2_ adsorbed volume per gram of material at standard temperature and pressure (STP = 273.15 K and 101.325 kPa). **d** Pore size distribution of modified Co/Mn ZIF shown as the derivative of pore volume to pore diameter (d*V*_p_/d*d*_p_) through the Barret–Joyner–Halenda (BJH) plot analysis with inset highlighting micropore (<2 nm) characteristic of type I materials.

The physicochemical properties of the fabricated Co/Mn ZIF materials were characterized prior to their application in *E. coli* sensing. First, X-ray diffraction (XRD) spectroscopy was employed to evaluate the crystallinity. X-ray diffractograms (Fig. 1a) revealed distinct peaks at 2*θ* of 7.44°, 10.5°, 12.82°, 14.76°, 16.48°, 18.1°, 24.26°, 24.62°, 26.66°, 29.66°, 30.58°, and 32.34°, corresponding to (011), (002), (112), (022), (013), (222), (114), (233), (134), (044), (244), and (235) crystal planes for both pristine ZIF-67 and Co/Mn ZIF of the selected Co/Mn ratios of 10:1, 5:1, 2:1, and 1:1. The patterns exhibit a topology consistent with the sodalite crystal framework (crystallography open database (COD) ID: 7222297, Supplementary Data 1^51,52^) of ZIF-67 indicating that Mn^2+^ incorporation does not introduce new peaks in the host^19,53^. However, the outset graph of Fig. 1a reveals a shift of the (011) and (112) peaks, indicating an Mn^2+^-driven reconstruction with Co^2+^. This shift occurs towards higher 2*θ* angles as the Mn^2+^ concentration increases to Co/Mn = 5:1^27,54^. Further increasing Mn^2+^ content (Co/Mn ZIF 2:1 and 1:1) resulted in a peak shift back to lower angles, attributed to lattice expansion due to a larger atomic radius of Mn^2+^ compared to Co^2+^^55,56^. Following Bragg’s law (Eq. (1))^57^, the right shift of the (011) peak from 7.19° (ZIF-67) to 7.27° (Co/Mn ZIF 10:1), and 7.40° (Co/Mn ZIF 5:1) corresponds to a decrease in the interplanar *d* spacing from 12.27 Å to 12.14 Å, and 11.92 Å, respectively. Conversely, a left shift of the peak to 7.31° (Co/Mn ZIF 2:1) and 7.26° (Co/Mn ZIF 1:1) indicates an increase in *d* spacing to 12.07 Å and 12.14 Å (see Table 1)^58^. These observations suggest partial Mn^2+^ incorporation into the lattice, as the shifts reflect competing effects of initial lattice contraction at lower Mn^2+^ concentration, followed by expansion at higher Mn^2+^ ratios, consistent with its larger ionic radius compared to Co^2+^^59,60^.

**Table 1:** X-ray diffraction (XRD) analysis of various electrode materials

Parameter	ZIF-67	Co/Mn ZIF 10:1	Co/Mn ZIF 5:1	Co/Mn ZIF 2:1	Co/Mn ZIF 1:1
2*θ* (°)	7.19	7.27	7.40	7.31	7.26
*d* (Å)	12.27	12.14	11.92	12.07	12.16

The calculated *d* spacing values of the (011) plane (using Eq. (1)) of ZIF-67 and Co/Mn ZIF with various Co/Mn ratios according to the respective measured 2*θ* angles.

Second, following XRD analysis, Fourier-transform infrared (FTIR) spectroscopy was employed to identify functional groups relevant to surface modification. As shown in Fig. 1b, FTIR spectra of bare ZIF-67 and Co/Mn ZIF exhibit a band at ~426 cm⁻¹ corresponding to the Co–N vibration (420–450 cm⁻¹ range)^61^, while the ~1143 cm⁻¹, ~1304 cm⁻¹, and ~1422 cm⁻¹ bands are attributed to the vibrations of C–N and C=C stretching, consistent with the aromatic amine range (1266–1440 cm⁻¹)^62,63^. The region between 600 and 1500 cm⁻¹ arises from imidazole ring modes, while the bands at 2840–3100 cm⁻¹ are attributed to C–H stretching^53,64^. The abundance of aromatic chains creates a hydrophobic environment, facilitating van der Waals interactions^65^. Moreover, these aromatic groups offer surface modification sites that enhance adsorption capacity and increase the accessible surface area^66,67^. Notably, the FTIR spectra of Co/Mn ZIF remain nearly identical across all concentration ratios, confirming that Mn^2+^ incorporation does not introduce a new functional group^68^, further supporting the XRD findings of partial lattice integration rather than phase formation.

Third, to investigate the surface properties of the modified Co/Mn ZIF, N_2_ adsorption-desorption measurements  and the specific surface area was calculated using the Brunauer–Emmett–Teller (BET) method. Figure 1c demonstrates the type I isotherm, which is typical for microporous material with a large N_2_ adsorbed volume at low relative pressure, revealing that modified Co/Mn ZIF has a higher specific surface area than the pristine ZIF-67 (*S*_BET_ = 1583 m² g^–1^). The Co/Mn ZIF 1:1 sample exhibits the largest surface area (*S*_BET_ = 2025 m² g^–1^), followed by the Co/Mn ZIF 2:1, 10:1, and 5:1 samples with *S*_BET_ = 1724, 1702, and 1647 m² g^–1^, respectively. Interestingly, the modified Co/Mn ZIF 5:1 (1647 m² g^–1^) exhibits the lowest increase in surface area, corresponding to the smallest *d* spacing value (see Table 1). A reduced *d* spacing likely results from the more efficient packing of atoms or ions within the crystal lattice, leading to denser packing with fewer exposed sites on the surface, reducing the amount of accessible surface area^69^. These values exceed other cobalt-based bimetallic materials^70–72^.

Furthermore, the total pore volume reaches its maximum value of 0.86 cm^3^ g^–1^ in the Co/Mn ZIF 1:1 sample, corresponding to the highest specific surface area. In comparison, pristine ZIF-67 has a total pore volume of 0.70 cm^3^ g^–1^. As Mn^2+^ concentration increases, the total pore volumes for the Co/Mn ZIF 10:1, 5:1, and 2:1 samples are 0.75, 0.73, and 0.76 cm^3^ g^–1^, respectively. The total pore volume is the cumulative pore volume per unit mass, calculated from adsorption data at a relative pressure close to 1 (i.e., *p/p*_0_ = 0.99). It corresponds to the integrated area under the d*V*_p_/d*d*_p_ curve across all pore diameters, as determined using BJH plot analysis in Fig. 1d. Figure 1d (inset) highlights the micropore size distribution of the modified Co/Mn ZIF as determined by micropore plot analysis. The pore diameters range from 0.42 to 2.7 nm, in which most of the pores are between 1 and 2 nm large. Pores having a diameter of <2 nm are classified as micropores according to the International Union of Pure and Applied Chemistry (IUPAC) standards^73^. However, this microporosity significantly affects the surface area of the material, as it provides abundant adsorption sites for molecular attachment. Micropores predominate the adsorption process, as revealed by the significant adsorption capacity observed at comparatively low pressure (*p/p*_0_ < 0.1)^74^.

**Fig. 2:**
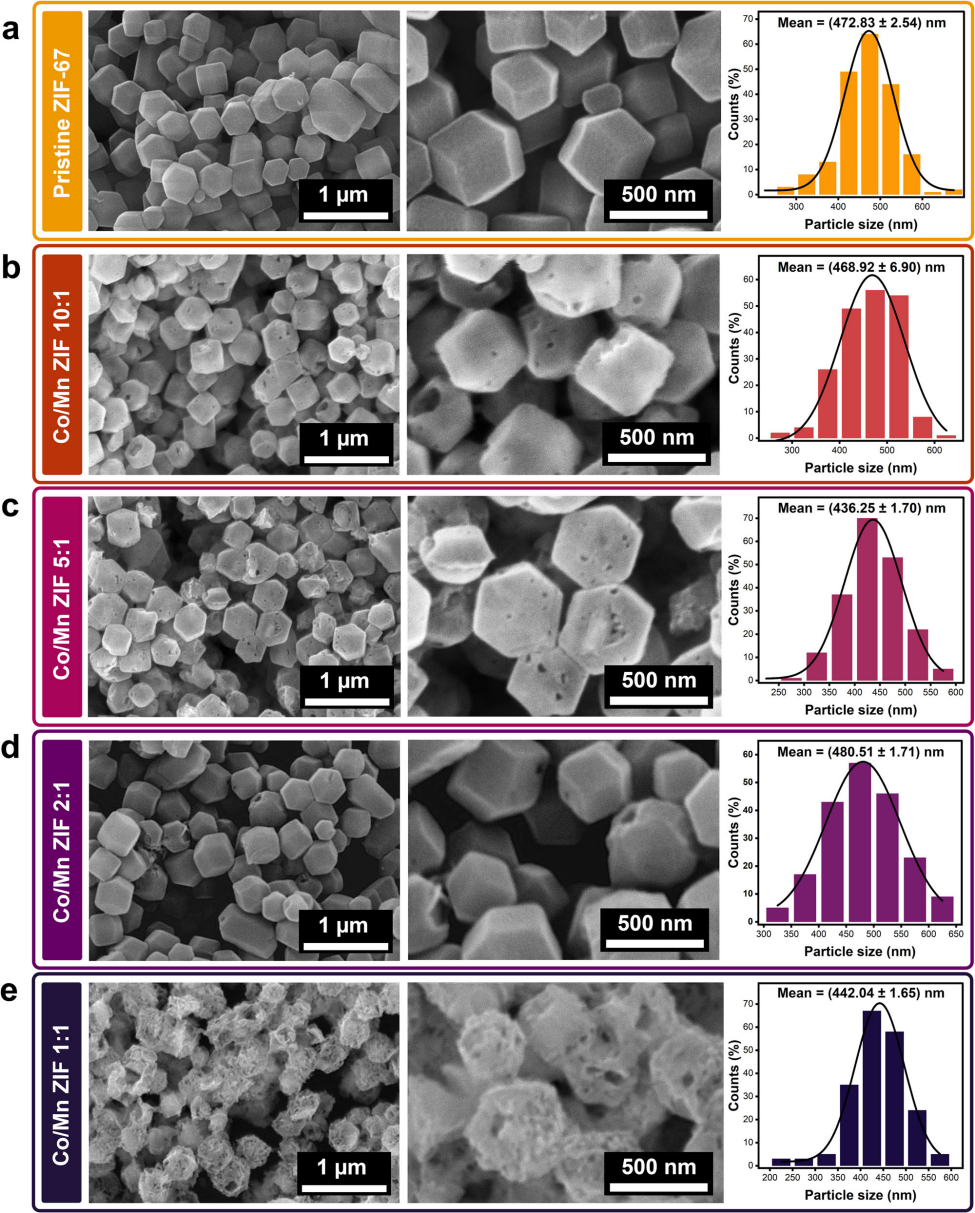
Morphologies and size distributions of modified Co/Mn ZIF materials with different concentration ratios. Scanning electron microscopy (SEM) images with two different magnifications (30000× and 80000×) and particle size distribution of **a** pristine ZIF-67 showing sharp rhombic dodecahedral, **b** Co/Mn ZIF 10:1 (pores arise when Mn^2+^ is added even in minor concentration), **c** Co/Mn ZIF 5:1, **d** Co/Mn ZIF 2:1 (structures remain sharp), and **e** Co/Mn ZIF 1:1 (material undergoes significant structural changes).

In these physicochemical structural attributes, the fourth characterization is scanning electron microscopy (SEM) analysis (see Fig. 2a–e), revealing that all Co/Mn ZIF samples having different concentration ratios retain the rhombic dodecahedral morphology of pristine ZIF-67 (see Fig. 2a)^75^. The particle size distribution was obtained by the Gaussian fitting according to the SEM images shown in Supplementary Fig. 1 on two spots taken at a magnification of 5000× (*n*_measurements_ = 100, each spot). The values are given as mean ± standard error (SE) of the fitted parameter obtained from the covariance matrix in Fig. 2a–e, and standard deviation (SD) from *n*_measurements_ = 200 are mentioned in this discussion. Figure 2a–e shows that the average particle size was determined from the peak center, ranging from 214 to 672 nm for Co/Mn ZIFs, consistent with the reported ZIF-67 dimensions (228 nm to 5.2 μm)^76^. The Co/Mn ZIF 10:1 sample showed slightly reduced particle size (468.92 ± 6.90 nm) (SD = 60.31 nm) compared to pristine ZIF-67 (472.83 ± 2.54 nm) (SD = 64.99 nm) (see Fig. 2a, b), along with rougher surfaces and sharper edges, indicating initial pore formation even at low Mn^2+^ concentration (see Fig. 2b). Progressive morphological changes became evident at higher Mn^2+^ ratios (Co/Mn ZIF 5:1 and 2:1) samples with the particle sizes of (436.25 ± 1.70 nm) (SD = 54.24 nm) and (480.51 ± 1.71 nm) (SD = 67.20 nm) (see Fig. 2c, d) displaying increased surface roughness, suggesting framework distortions that enhanced porosity. The structure exhibits high disorder at the highest Co/Mn ZIF 1:1 ratio, with high surface roughness and significant structural changes (442.04 ± 1.65 nm) (SD = 62.25 nm) (see Fig. 2e). The incorporation of Mn²⁺ improves pore formation due to its higher ionic radius compared to Co²⁺. The valence state dictates the spatial arrangement of the organic ligands, which in turn defines the pore shape and the framework’s size^77^. Charge balance during Mn²⁺ substitution into ZIF-67 may also introduce defects or vacancies, further modifying porosity^78^. The observed structural evolution correlates well with the BET results, where increasing Mn²⁺ content leads to greater surface area and pore volume.

Mn content was quantitatively evaluated by X-ray fluorescence (XRF) spectroscopy. Table 2 indicates that the corresponding Mn/(Co+Mn) molar ratio continuously ascended from 0.0011 (Co/Mn ZIF 10:1) to 0.0098 (Co/Mn ZIF 1:1), respectively. This trend reflects a controlled introduction of Mn into the synthesis process of the ZIF-67 framework, further supported by an inverse correlation in the elemental weight percentages of Co and Mn. The observed trend follows the intended stoichiometric ratios, despite the Mn content being found at a comparatively low rate. Mn^2+^ preferentially utilizes defect sites instead of uniformly substituting Co^2+^ within the lattice^79^.

**Table 2:** Elemental atomic composition of Co/Mn ZIF materials on X-ray fluorescence (XRF) spectroscopy

Samples	Elemental atomic composition (wt%)		Mn/(Co + Mn) molar ratio
	Co	Mn	
ZIF-67	(100.00 ± 0.09)	Not detected	0
Co/Mn ZIF 10:1	(99.90 ± 0.08)	(0.10 ± 0.003)	(0.0011 ± 0.00003)
Co/Mn ZIF 5:1	(99.79 ± 0.36)	(0.21 ± 0.02)	(0.0022 ± 0.0002)
Co/Mn ZIF 2:1	(99.53 ± 0.37)	(0.47 ± 0.02)	(0.0050 ± 0.0002)
Co/Mn ZIF 1:1	(99.08 ± 0.08)	(0.92 ± 0.01)	(0.0098 ± 0.0001)

The Mn/(Co + Mn) molar ratio incorporated in the ZIF-67 framework ascended from (0.0010 ± 0.00003) in Co/Mn ZIF 10:1 to (0.0098 ± 0.0001) in Co/Mn ZIF 1:1. Values represent mean ± standard deviation (SD) (1σ, the internal precision of the instrument).

### Electrochemical properties of Co/Mn ZIF electrodes

In addition to physicochemical characterization, the electrochemical analysis revealed enhanced electron transfer properties of the modified screen-printed carbon electrode (SPCE/Co/Mn ZIF). Before conducting detailed CV measurements, two electrolyte systems-0.01 M phosphate buffer saline (PBS) and mixed 5 mM potassium ferricyanide (K_3_[Fe(CN)]_6_) with 0.1 M potassium chloride (KCl) in 0.01 M PBS-were tested to check whether the SPCE/Co/Mn ZIF electrode was electrochemically active or passivated through a scan rate (*V*) of 20 mV s^–1^ and potential range from −0.5 to 1.0 V. As shown in Supplementary Fig. 2a, a distinct and reversible peak current (*I*_p_) corresponding to the hexacyanoferrate (III)/(II) ([Fe(CN)_6_]^3–^/^4–^) redox couple was observed in the mixed electrolyte, while a broad peak was present in PBS alone. It is plausible that phosphate anions in PBS could interact with the metal centers, potentially facilitating redox activity through a synergistic effect between two metals^80,81^, and the phosphate as a typical solid-state electrocatalyst^82–86^. Nevertheless, this broad peak remains less pronounced than the well-defined [Fe(CN)_6_]^3–^/^4–^ peaks. The presence of a broad peak demonstrates that the Co/Mn ZIF electrode facilitates efficient electron transfer when an external redox probe, like [K_3_Fe(CN)_6_], is introduced.

Following this evaluation, various concentrations of mixed K_3_[Fe(CN)]_6_ were prepared with 0.1 M KCl in 0.01 M PBS and the SPCE/Co/Mn ZIF electrode was evaluated through a scan rate of 20 mV s^–1^ and potential range from –0.5 to 1.0 V. Supplementary Fig. 2b demonstrates an increasing *I*_p_ of the redox reaction occurred by increasing the concentration of K_3_[Fe(CN)]_6_ electrolyte. The electroactive surface area was calculated based on the Randles–Ševčík equation (Eq. (2)), with the highest value at a concentration of 5 mM (0.19 cm^2^). In comparison, lower and higher concentrations yielded reduced electroactive surface areas, i.e., 1 mM (0.04 cm^2^), 10 mM (0.13 cm^2^), and 15 mM (0.12 cm^2^). A high electroactive surface area improves the electrochemical efficiency by providing more available sites for redox reaction, which boosts the current response^87^. The drop in the other concentration’s electroactive surface area suggests a deviation from the optimal diffusion-controlled behavior, brought by mass transport limitations or capacitive distributions^88^. Based on this observation, 5 mM K_3_[Fe(CN)]_6_ electrolyte was selected as the optimal concentration for subsequent electrochemical measurements. Although concerns over the toxicity of ferricyanide exist, the application of 5 mM of K_3_[Fe(CN)]_6_ electrolyte is well-supported by several previous studies^43–46^.

**Fig. 3:**
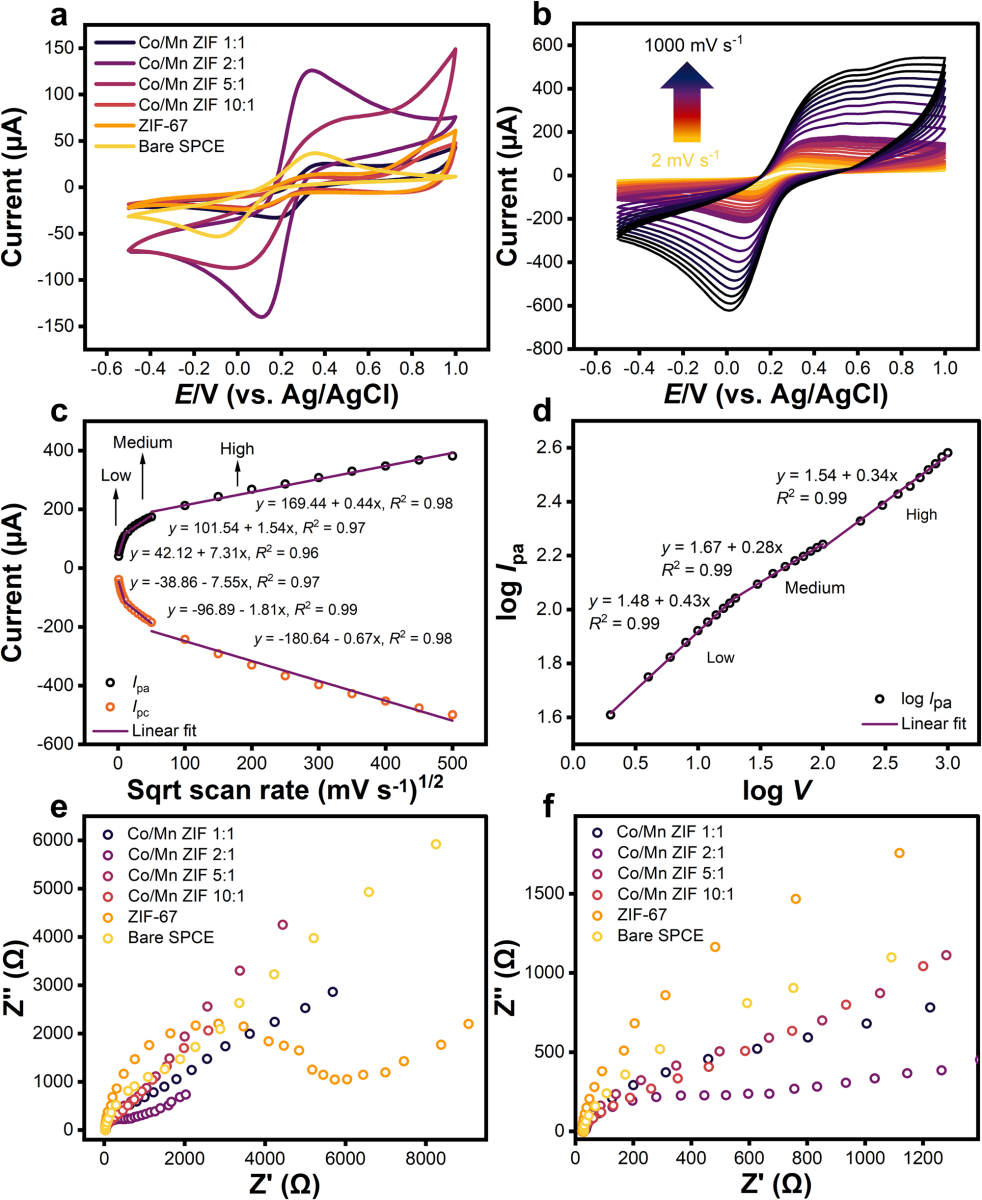
Electrochemical characterization of screen-printed carbon electrode (SPCE/Co/Mn ZIF) electrodes in 5 mM potassium ferricyanide (K_3_[Fe(CN)_6_]) with 0.1 M potassium chloride (KCl) in 0.01 M phosphate buffer saline (PBS). **a** Cyclic voltammetry (CV) curves of hexacyanoferrate(III)/(II) [Fe(CN)_6_]^3–^/^4–^ redox reaction at 20 mV s^–1^ scan rate (*V*) showing enhanced current response for Co/Mn ZIF 2:1 (purple) compared to other ratios. **b** CV curves recorded at a scan rate of 2 to 1000 mV s^–1^, demonstrating increased anodic peak current (*I*_pa_) due to the increase of scan rate. **c** Linear correlation of *I*_p_ vs. square root (sqrt) scan rate (*V*^1/2^) across three regions of low (2–20 mV s^–1^), medium (20–100 mV s^–1^), and high (100–1000 mV s^–1^) scan rates with dependence of *R*^2^ value close to ≈1 (0.96, 0.97, 0.98 for *I*_pa_ and 0.97, 0.99, 0.98 for cathodic peak current (*I*_pc_), respectively). **d** Log–log plot of *I*_pa_ vs. *V*, showing a slope of 0.43 (close to ≈0.5) at low scan rate, confirming diffusion-controlled, while medium and high scan rates demonstrate lower slopes (0.23 and 0.34), respectively, indicating a deviation from ideal diffusion behavior. **e** Electrochemical impedance spectroscopy (EIS) measured at a frequency range between 10^–2^ and 10^6 ^Hz, revealing minimal charge transfer resistance (*R*_ct_) for Co/Mn ZIF 2:1, and **f** an enlarged view of the low impedance region for better visualization of *R*_ct_ variations among the Co/Mn ZIF electrodes.

Figure 3a provides the CV curve of [Fe(CN)_6_]^3–^/^4–^ redox reaction, demonstrating that the modified SPCE/Co/Mn ZIF 2:1 electrode performance is enhanced through anodic peak current (*I*_pa_) (124 µA), which is higher than measured with the pristine SPCE/ZIF-67 electrode (13.6 µA). Its performance exceeds other modified SPCE/Co/Mn ZIF and other bimetallic MOF-based SPCE electrodes (e.g., Fe^III^-HMOF-5, NiCo MOF, and CoNi MOF) (see Supplementary Table 1)^89–91^. According to the Randles–Ševčík equation (Eq. (2)) this optimal ratio also demonstrated a larger electroactive surface area of 0.24 cm^2^ vs. 0.03 cm^2^ for ZIF-67, suggesting efficient Mn^2+^ incorporation that enhances electron transfer pathways and increased surface roughness at the electrode interface while maintaining structural integrity, consistent with the SEM image (see Fig. 2d). The electrochemical result indicates the controlled Mn^2+^ incorporation at the 2:1 ratio creates an ideal balance between defect generation and charge transport properties for biosensing applications^92^.

Figure 3b demonstrates the scan rate dependence in the modified SPCE/Co/Mn ZIF 2:1 electrode from 2 mV s^–1^ to 1000 mV s^–1^, performing a quasi-reversible behavior with symmetric anodic/cathodic peak current (*I*_pa_/*I*_pc_) ≈ 1 (see Supplementary Table 2). The observed potential peak separation (*ΔE*_p_ = 180–510 mV) is significantly larger than its theoretical value of 59 mV, representing the thermodynamic and kinetic relationship for a single-electron transfer reaction under ideal conditions at room temperature^93^. This suggests a slow electron transfer process that is beneficial in biosensing applications since biosensors should enable stable detection across a wide range of analyte concentrations, leading to a gradual system response with reduced susceptibility to interference, thereby enhancing selectivity^94,95^. Furthermore, a linear relationship of *I*_p_ vs. square root (sqrt) scan rate (*V*^1/2^) confirms electron transfer is diffusion-controlled across all scan rate regions-low (2–20 mV s^–1^), medium (20–100 mV s^–1^), and high (100–1000 mV s^–1^)-with only slight variations in *R*^2^ value fit close to ≈1 (0.96, 0.97, 0.98 for *I*_pa_ and 0.97, 0.99, 0.98 for *I*_pc_, respectively) confirm the assumption of linearity (see Fig. 3c). To further evaluate the nature reaction process, we constructed a log–log plot of *I*_pa_ vs. *V* (Fig. 3d). In this approach, a slope near ≈1 indicates adsorption–controlled, while a slope close to ≈0.5 indicates diffusion-controlled^96–98^. Figure 3d shows a slope value of 0.43 at the lowest scan rate region (2–20 mV s^–1^). In contrast, medium and high regions performed lower slope values of 0.28 and 0.34, respectively, indicating a deviation from ideal diffusion behavior. The decrease in slope with increasing scan rate is likely due to rising kinetic constraints from limited ion accessibility or slower surface reactions^99^. Additionally, the reduced response at high scan rate may be associated with partial saturation of active sites, where the rate of ion transport cannot keep up with the scan rate, and the ions do not have adequate time to diffuse deeply into the structure. This limitation may lead to the appearance of shoulder peaks or peak broadening at high scan rate, as observed in Fig. 3b^100^. This observation led to the selection of the lowest region of scan rate (2–20 mV s^–1^) for the subsequent differential pulse voltammetry (DPV) measurement.

**Fig. 4:**
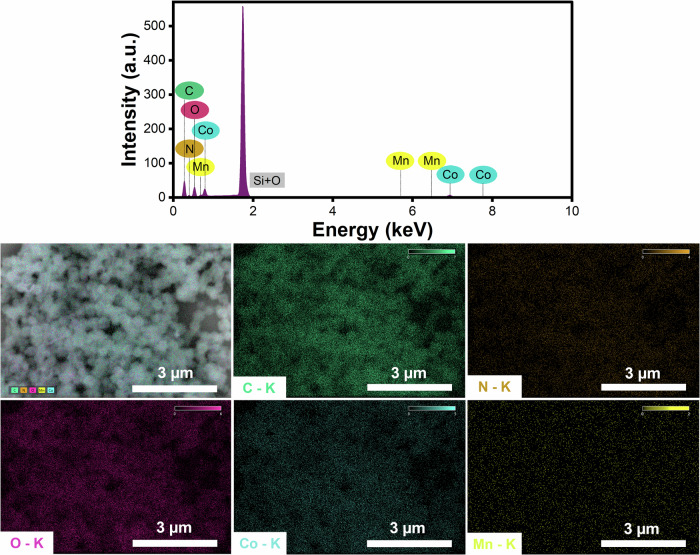
Energy-dispersive X-ray spectroscopy (EDS) of Co/Mn ZIF 2:1 sample. EDS mapping combined with field-emission scanning electron microscopy (FE-SEM) to demonstrate clear Co distribution and detectable Mn signal.

Electrochemical impedance spectroscopy (EIS) characterization was performed to quantify charge transfer kinetics and determine the ideal Co/Mn ratio. Figure 3e displays the Nyquist plots revealing distinct semicircular regions corresponding to the charge transfer resistance (*R*_ct_), with an enlarged view presented in Fig. 3f to better visualize the *R*_ct_ differences among Co/Mn ZIF electrodes (the fitted values detailed in Supplementary Table 3). The *R*_ct_ was determined by subtracting the solution resistance (*R*_1_), obtained from the high-frequency intercept of the semicircle, from the total resistance at low frequency (*R*_2_). The lowest *R*_ct_ value of 322 Ω was obtained for Co/Mn ZIF 2:1 electrode, indicating enhanced kinetics and the most efficient electron transfer among the selected Co/Mn ratios, and supporting the CV test results^101^. The *R*_ct_ demonstrates a non-linear trend as a trade-off between the conductivity initiated by Mn-related defects and the structural integrity of the composite. This is reinforced by the elemental mapping using energy-dispersive X-ray spectroscopy (EDS), indicating the presence of Mn in the Co/Mn ZIF 2:1 sample, though the distribution appears sparse due to its low content (see Table 2). Nevertheless, the corresponding EDS spectrum confirms that the Mn signal is distinguishable above the background (see Fig. 4). The enhancements observed in both CV and EIS measurements, along with the material’s high surface area (1724 m^2^ g^–1^) and retained polyhedral morphology, underscore the Co/Mn ZIF 2:1 composite as the optimal platform for *E. coli* biosensor development, offering simultaneous enhancements in electron transfer efficiency and mass transport capabilities.

### Structural hydrostability of Co/Mn ZIF 2:1

To ensure the aqueous endurance, the hydrostability of Co/Mn ZIF 2:1 was examined after seven days of water incubation. A gradual color change from bright to dark purple was observed, suggesting a marginal change in the coordination of the metal centers, potentially related to the ligand local symmetry (see Supplementary Fig. 3)^102,103^. XRD analysis results (Supplementary Fig. 4 and Supplementary Table 4) show that the main crystal plane before seven days of water incubation (Fig. 1a) remained detectable, and its corresponding (011) *d* spacing was largely unchanged, from 12.07 to 12.04 Å. However, the peak area under the (011) plane was also lowered (from 1456.18 to 574.82), providing a quantitative measure of the significant loss of crystallinity. This confirms that a substantial portion of the material has undergone partial degradation and may transform to the amorphous phase^104,105^. These findings suggest minor reordering in the core crystal structure, while a significant portion of the material loses crystallinity, as is often observed in ZIFs exposed to an aqueous environment.

### Biosensor performance towards *Escherichia coli*

**Fig. 5:**
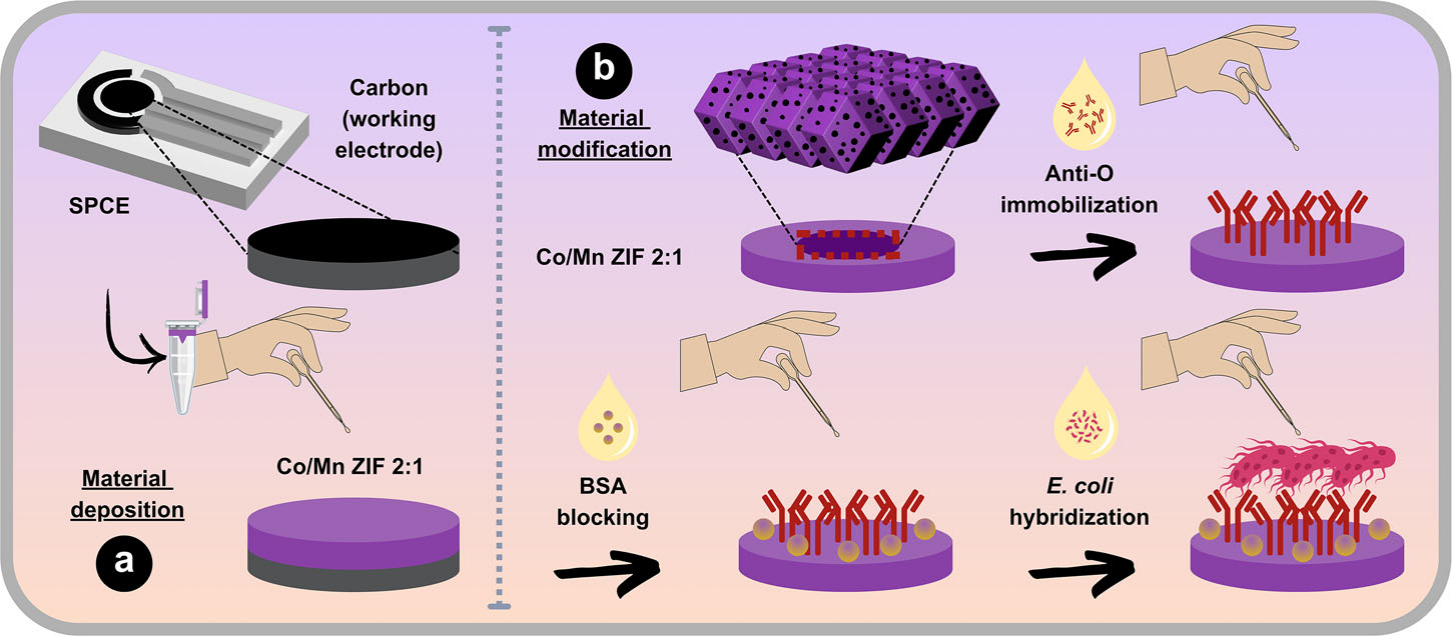
Material surface modification procedures for *E. coli* sensing. **a** The Co/Mn ZIF 2:1 material deposition process (SPCE/Co/Mn ZIF 2:1 electrode) followed by **b** step-by-step material modification on deposited Co/Mn ZIF 2:1 through anti-O antibody immobilization (SPCE/Co/Mn ZIF 2:1/anti-O electrode), BSA non-target blocking (SPCE/Co/Mn ZIF 2:1/anti-O/BSA electrode), and *E. coli* hybridization for sensor testing (SPCE/Co/Mn ZIF 2:1/anti-O/BSA/*E. coli*).

After the optimized Co/Mn ZIF 2:1 material composition had been determined, further surface modifications were conducted to improve the physicochemical performance and selectivity of the Co/Mn ZIF 2:1 electrode to detect *E. coli*. These include immobilizing anti-O antibodies through physical adsorption and bovine serum albumin (BSA) to block non-specific areas, after depositing Co/Mn ZIF 2:1 on the working electrode (see Fig. 5). Several evaluation methods (i.e., water contact angle (WCA), attenuated total reflectance-Fourier transform infrared (ATR-FTIR) spectroscopy, and CV measurements) were used to verify the physicochemical and electrochemical changes.

WCA measurements shown in Supplementary Fig. 5a–d reveal distinct wettability changes across the functionalization steps, representing values of mean ± SD, *n*_measurements_ = 5. The working electrode (bare SPCE) that initially has a WCA of (94.50 ± 0.40)° has become more hydrophobic after Co/Mn ZIF 2:1 deposition (142.30 ± 0.70)° (see Supplementary Fig. 5a, b), attributed to the outward orientation of 2-methylimidazole hydrophobic CH_3_ groups^106–108^ and air pocket formation in micropores^109^, which enhances the lotus effect^110,111^. Anti-O antibody immobilization reduces hydrophobicity (WCA of (121.10 ± 1.20)°) (see Supplementary Fig. 5c), performing competing interfacial effects that the Fab region of the antibody exposes polar residues for antigen binding, while the Fc domain CH_2_/CH_3_ of the antibody maintains hydrophobic anchoring to the ZIF via van der Waals interactions^112,113^. The WCA decreases significantly to (97.80 ± 0.90)° upon BSA blocking, demonstrating a shift to hydrophilic behavior (see Supplementary Fig. 5d). It is ascribed to hydrophilic amino acid residues in BSA that form structured hydration layers^114^. This improved wettability ensures optimal biosensor performance in aqueous environments.

**Fig. 6:**
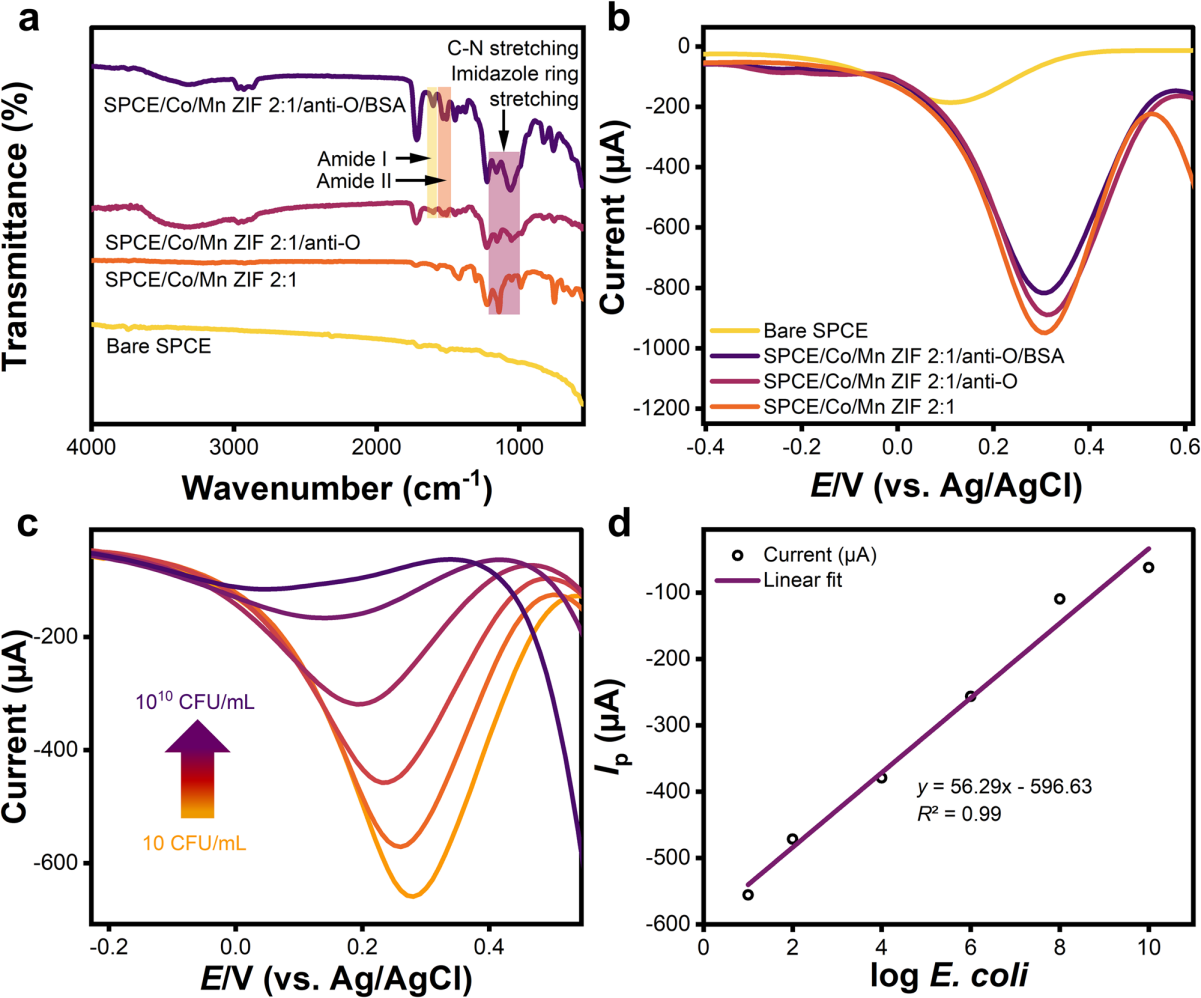
Electrode surface modification and its sensitivity tests. **a** Attenuated total reflectance-Fourier transform infrared (ATR-FTIR) spectra exhibiting the amides II and I at 1506–1602 cm^–1^ after anti-O antibody immobilization and BSA blocking. **b** Differential pulse voltammetry (DPV) curves on the step-by-step anti-O antibody immobilization showing a decrease in reduction peak current after layer-by-layer stacking. **c** DPV curves for different concentrations of *E. coli* (10, 10^2^, 10^4^, 10^6^, 10^8^, and 10^10^ CFU mL^–1^). **d** Increasing analyte concentration leads to a less negative reduction peak current, which increases linearly with the logarithm of concentration.

ATR-FTIR spectra (Fig. 6a) confirm the stepwise biofunctionalization of electrode surfaces. The SPCE/Co/Mn ZIF 2:1 spectrum maintains its characteristic fingerprint region (1000–1200 cm⁻¹, C-N stretching from 2-methylimidazole) after the deposition onto the working electrode of SPCE, matching the previous investigation on its powder phase (see Fig. 1b). Anti-O antibody immobilization induces two spectral changes. The first change is the broadening of C-N stretching (1000–1200 cm⁻¹), suggesting distortion of imidazole ring vibrations due to anti-O antibody physical adsorption within ZIF pores^115,116^. The second alteration is the emergence of amide II (1506–1541 cm⁻¹, N-H bending and C-N stretching) and amide I (1602 cm⁻¹, C=O stretch) bands, verifying protein presence^117,118^. Subsequent BSA blocking amplifies these amide signals, further confirming the immobilization via hydrophobic interactions (Fc region anchoring) without covalent bond formation^119^. The existence of this amide is also related to the hydrophilic state of the surface material, orienting the protein by exposing the amide group to the environment. As a result, hydrogen bonding with water molecules on the surface may be improved.

DPV analysis was performed to evaluate the surface modification steps using an electrolyte containing 5 mM of K_3_[Fe(CN)_6_] with 0.1 M KCl in 0.01 M PBS at a potential range of –0.5 to 1.0 V and a scan rate of 20 mV s^–1^. The attenuation of the reduction peak currents (see Fig. 6b) quantitatively validated successful antibody immobilization, revealing reduced electron transfer kinetics due to surface blocking^120,121^. For *E. coli* detection, DPV responses exhibited concentration-dependent decrease in peak current across the range of 10–10^10^ CFU mL^–1^ (see Fig. 6c). The *E. coli* was detected through its cell wall containing lipopolysaccharides with the O-polysaccharides as the specific identity of *E. coli* occupying about 75% of the surface area of the bacteria^122^. Figure 6d presents the calibration curve, revealing a linear relationship (reduction peak current vs. log *E. coli*; $y=56.29x-596.93$, *R*^2^ = 0.99), with a calculated limit of detection (LoD) of 1 CFU mL^–1^ (Eq. (3), SD of blank responses (*S*_b_) = 19.67, *n*_measurements_ = 3)^123^. The developed biosensor outperforms MOF-based optical-electrochemical counterparts (see Table 3) in both detection range (10–10^10^ CFU mL^–1^) and sensitivity, while avoiding the complexity and cost of an optical setup^38–46^.

**Table 3:** Performance comparison of MOF-based biosensors for *E. coli* detection

Material	Receptor	Transducer	Detection range (CFU mL^–1^)	LoD (CFU mL^–1^)	Reference
Tb-BTC	Antibody	Optical	1.3 × 10^2^–1.3 × 10^8^	3	^38^
NH_2_-MIL-101 (Fe) MOF	Bacteriophage	Optical	5.78 × 10^1^–5.78 × 10^6^	652	^39^
Zirconium MOF (UiO-66)	DNA	Optical	1.3 × 10^2^–6.5×10^4^	40	^40^
PCN-223-Fe (AgPt/PCN-223-Fe	Antibody	Optical	10^3^–10^8^	276	^41^
Cu-MOF NPs	Aptamer	Optical	16–1.6 × 10^6^	2	^42^
Cu_3_(BTC)_2_-PANI	Antibody	Electrochemical	2.0–2 × 10^8^	2	^43^
MOF MIL 53/PEDOT: Polystyrene	Antibody	Electrochemical	2.1 × 10^2^–2.1 × 10^8^	4	^44^
Cu_3_(BTC)_2_-PANI	Aptamer	Electrochemical	2.1 × 10^1^–2.1 × 10^7^	2	^45^
CdS@ZIF-8 MOF	Antibody	Electrochemical	10–10^8^	3	^46^
Co/Mn ZIF	Antibody	Electrochemical	10–10^10^	1	This work

Two different transduction methods (i.e., optical and electrochemical transducers) have been mainly used for biosensors.

**Fig. 7:**
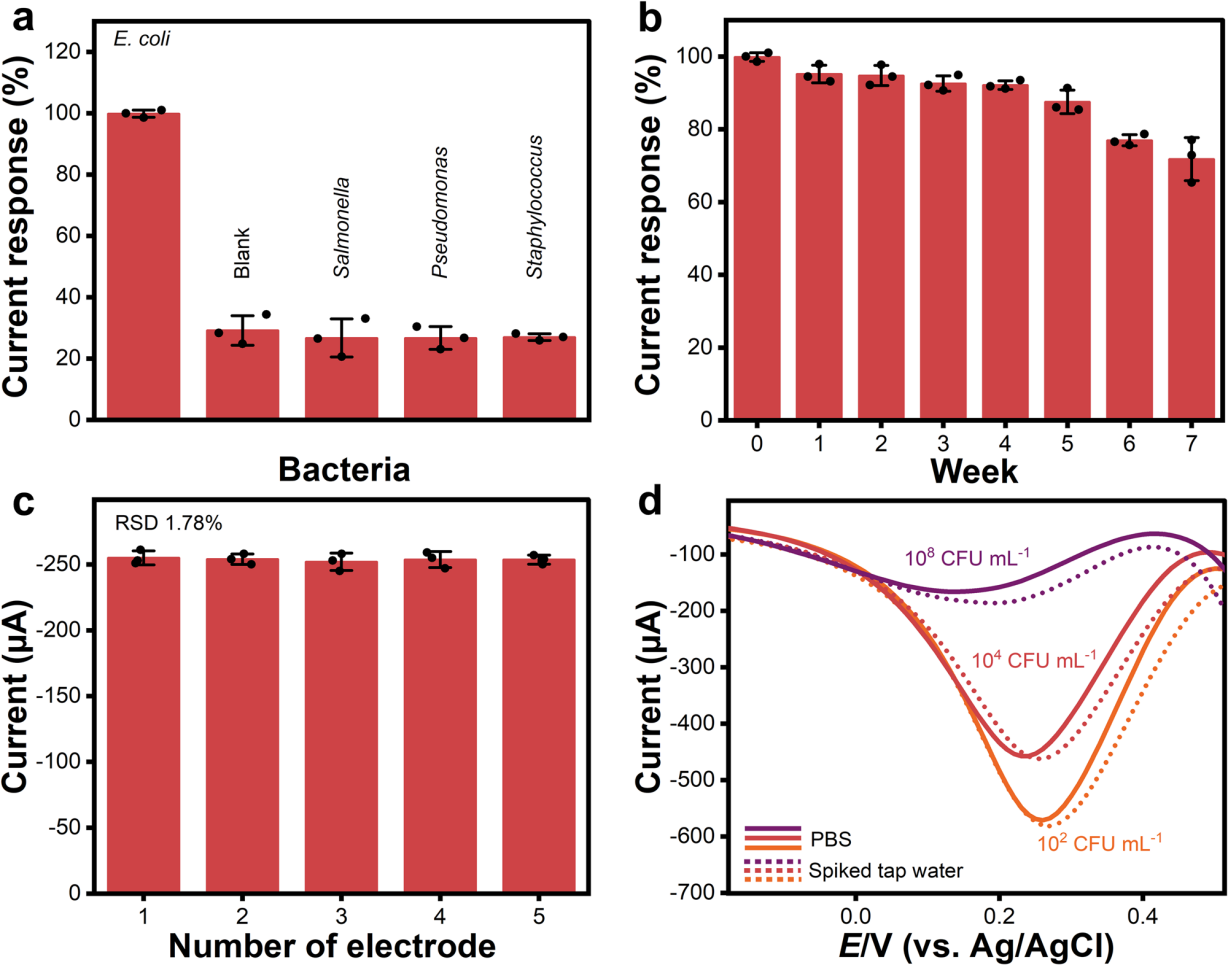
Evaluation metrics of SPCE/Co/Mn ZIF 2:1/anti-O/BSA biosensor performance towards *E. coli* and nontarget bacteria. **a** Selectivity test results showing sensor responses (%) toward *E. coli*, other non-specific bacteria (*Salmonella, Pseudomonas*, and *Staphylococcus* (10^6^ CFU mL^–1^)), and blank (PBS 0.01 M). **b** Stability test results exhibiting sensor responses (%) from SPCE/Co/Mn ZIF 2:1/anti-O/BSA electrode against 10^6^ CFU mL^–1^ of *E.coli* after they had been stored over a storage period of up to 7 weeks. **c** Reproducibility test results showing sensor responses (µA) from different SPCE/Co/Mn ZIF 2:1/anti-O/BSA electrodes fabricated from the same batch. Data points (black dots) for selectivity, stability, and reproducibility results are exhibited with the error bars corresponding to one SD (1σ). **d** Recovery response (DPV curve) of SPCE/Co/Mn ZIF 2:1/anti-O/BSA electrode towards *E. coli* spiked (10^2^, 10^4^, and 10^8^ CFU mL^–1^) in tap water compared to 0.01 M PBS.

Further evaluations of biosensor performance were applied to verify the selectivity, stability, reproducibility, and recovery of SPCE/Co/Mn ZIF 2:1/anti-O/BSA electrode. These evaluations were conducted through DPV measurements using an electrolyte containing 5 mM of K_3_[Fe(CN)_6_] with 0.1 M KCl in 0.01 M PBS at a potential range of –0.5 to 1.0 V and a scan rate of 20 mV s^–1^. A selectivity test was conducted against 10^6^ CFU mL^–1^ (in 0.01 M PBS) *Salmonella, Pseudomonas, Staphylococcus*, and the blank containing only 0.01 M PBS. All non-target bacteria exhibit minimal deviation from the baseline current in the selectivity test, remaining below 35% of the *E. coli* current response (SD, *n*_measurements_ = 3) (see Fig. 7a). Additionally, the blank sample showed a low current response (29.15 ± 4.83)% (see Supplementary Table 5), indicating that the biosensor was unaffected by external interference. These findings suggest that SPCE/Co/Mn ZIF 2:1/anti-O/BSA electrode can selectively detect *E. coli* over non-selective bacteria. For the long-term stability test, Fig. 7b shows the results of the stability assay based on a retention calculation (Eq. (4)). The SPCE/Co/Mn ZIF 2:1/anti-O/BSA-based biosensors for *E. coli* detection demonstrated good stability, maintaining over 80% functionality by week 5. This implies the SPCE/Co/Mn ZIF 2:1/anti-O/BSA electrode is reliable and stable for *E. coli* detection for up to one month, with only a gradual decrease in sensitivity. By averaging *n*_measurements_ = 3, the SD of the measured response was applied to compute the error bars complementing the data points (see Supplementary Table 6). It suggests consistent and reproducible data, with minimal fluctuation in stability over the testing period.

Furthermore, reproducibility tests were performed on five SPCE/Co/Mn ZIF 2:1/anti-O/BSA electrodes that were fabricated in the same batch and tested against 10^6^ CFU mL^–1^ of *E. coli*. Figure 7c depicts the excellent reproducibility of the five fabricated biosensors with a relative standard deviation (RSD) of 1.78% (*n*_measurements_ = 3 per electrode), encompassing all data points at all fabricated electrode batches (see Supplementary Table 7). In addition, a recovery test was performed by measuring the observed current response on the specific concentration (10^2^, 10^4^, and 10^8^ CFU mL^–1^) of *E. coli* spiked in tap water, in comparison to the obtained current on the use of standard 0.01 M PBS (Eq. (5))^124,125^. The percentage recovery shows satisfaction with the values close to 100% from (93.10 ± 2.61)% to (107.52 ± 5.90)% (*n*_measurements_ = 5), and the RSD was found to be <7% (see Fig. 7d and Supplementary Table 8). This combination of batch-to-batch reproducibility and reliable recovery in spiked samples validates the platform’s readiness for environmental monitoring applications where precision and accuracy are critical.

## Conclusions

The Co/Mn ZIF reveals considerable promise to be used in biosensing electrodes for detecting *E. coli*. The incorporation of Mn in ZIF-67 offers significant advantages (e.g., an extended surface area and enhanced electron transfer), leading to a notably high current response. By an effective selection and modification of anti-O antibodies, selectivity against other bacteria has been achieved. Differential pulse voltammetry (DPV) has demonstrated a linear reduction peak current vs. logarithm of analyte concentration dependence with *E. coli*, while also exhibiting long-term stability, an excellent antibody assay’s reproducibility, and a feasibility for application in real water detection through tap-water-based recovery tests. Owing to the possibility of using different types of surface functionalization, the Co/Mn ZIF composite can also be considered a promising base material for advancing other various biosensor technologies beyond *E. coli* detection.

## Methods

### Materials and chemicals

To synthesize the Co/Mn ZIF, cobalt nitrate hexahydrate (Co(No_3_)_2_·6H_2_O) (Sigma Aldrich) and manganese nitrate tetrahydrate (Mn(No_3_)_2_·4H_2_O) (Merck) were used as the metal ion source, coordinated to the ligand 2-methylimidazole (Sigma Aldrich). PBS from Bio Gear, was frequently utilized as a solvent (pH 7.4), along with a mixture of K_3_[Fe(CN)_6_]_,_ and KCl (Sigma Aldrich) to prepare an electrolyte solution. For surface modification, anti-O157 *E. coli* monoclonal antibody (MyBioSource MBS 530858) and BSA (Sigma Aldrich) were applied as the bioreceptor and the blocking agent, respectively. *Escherichia coli* (O157:H7) (ATCC 43895) was used as the target analyte. Meanwhile, *Salmonella enterica* serovar Typhimurium (ATCC 14028)*, Pseudomonas aeruginosa* (ATCC 9027), and *Staphylococcus aureus* (ATCC 25923) were employed as non-target or competing analytes relevant to clinical disease. For further functionalization and electrochemical measurements, SPCEs were obtained from Labotopia, Indonesia.

### Synthesis of ZIF-67 and Co/Mn ZIF

ZIF-67 was synthesized using the co-precipitation method^126^. To yield a metal-ion solution, 2 mmol of cobalt nitrate hexahydrate was dissolved in 30 mL of methanol. In a separate beaker, 8 mmol of 2-methylimidazole was dissolved in 30 mL of methanol for a ligand solution. After 15 min of stirring, the metal-ion and ligand solutions were mixed mildly. The resulting solution changed into purple soon after it was mixed, and it was agitated for 60 min afterward. For Co/Mn ZIF synthesis, manganese nitrate tetrahydrate was mixed into the metal-ion solution, ensuring that the Mn^2+^ content remained below (or equal to) that of Co^2+^. At a total metal precursor concentration of 2 mmol, different ratios of Co^2+^ and Mn^2+^ were designed to be 10:1, 5:1, 2:1, and 1:1. Following the typical method for the pristine ZIF-67 synthesis described above, the same following stages were also taken for Co/Mn ZIF synthesis.

### Structural characterization and analysis of Co/Mn ZIF

To investigate the effect of Mn^2+^ on the crystal phase of Co-based ZIF-67, advanced XRD (Bruker D8, Germany) was deployed. The crystallinity of the materials was assessed by comparing the experimental diffractogram with the simulated structure obtained from the COD ID 7222297^51,52^. Following Bragg’s Law (Eq. (1))^57^, the most intense diffraction peak was analyzed to determine the characteristic interplanar spacing (*d* spacing) of the crystals:

1 $${n}_{{\rm{order}}}\lambda =2d\sin \theta$$

To ensure that the reflected X-rays are within the practical measurement limits, *n*_order_ was selected 1 as the first-order diffraction. The incident X-ray wavelength (CuK_α_) radiation (*λ*) was set to 1.54 Å, while *θ* was measured from the angle of incidence relative to the crystal plane.

The chemical structure, including functional group and bonding interactions between metal ions and organic ligands of Co/Mn ZIF, was identified using FTIR (Prestige 21, Shimadzu, Japan) spectroscopy. Surface and pore properties (e.g., specific surface area, adsorption–desorption behavior, and pore size distribution) of Co/Mn ZIF, were analyzed using BET (Microtrac Belsorp MINI X, Japan). All samples were subjected to degassing at 150 °C for 12 h prior to the measurements. Furthermore, morphological and particle size analyses were performed using SEM (Hitachi SU-3500, Japan), followed by EDS (JEOL JIB-4610F, Japan) mapping to assess the spatial distribution of Co and Mn within the ZIF framework. Additionally, XRF spectroscopy (Orbis Micro, Australia) was also employed for the quantitative determination of elemental atomic composition.

### Electrode preparation of Co/Mn ZIF

The SPCE comprising three electrodes (counter and working (carbon-based material), and reference electrodes (Ag/AgCl)), were pre-treated through an activation process using 1 M H_2_SO_4_ in CV measurements with a scan rate of 100 mV s^–1^ and a potential range of 0–1.0 V. To start fabricating the electrode of an electrochemical sensor, a 500 µL material dispersion was prepared by dissolving a mixture of 2 mg Co/Mn ZIF, 375 µL of distilled water (aqua bidest), 100 µL of isopropanol, and 25 µL of Nafion 5% (Sigma Aldrich). Then, the mixture was sonicated to homogenize for 15 min. Following this, an activated SPCE was obtained by coating the working electrode’s surface layer with 10 µL of the mixture solution, which was labeled as SPCE/Co/Mn ZIF electrode and subjected to vacuum drying for 24 h before its use.

### Electrochemical characterization of SPCE/Co/Mn ZIF

CV and EIS were employed to evaluate the electron transfer kinetics at the electrode surface. Before these measurements, the CV performance of the SPCE/Co/Mn ZIF electrode was assessed using two different electrolyte systems: (i) 0.01 M PBS, and (ii) mixed 5 mM K_3_[Fe(CN)_6_] and 0.1 M KCl in 0.01 M PBS to check the electrochemical activity on the electrode surface. A PalmSens potentiostat (Netherlands) was used in these measurements with a scan rate of 20 mV s^–1^ throughout a potential range of –0.5 to 1.0 V. After knowing that the mixed electrolyte performed an optimum activity, various concentrations (1–15 mM) of mixed K_3_[Fe(CN)_6_] with 0.1 M KCl in 0.01 M of PBS were tested via CV measurements (scan rate of 20 mV s^–1^, potential range of –0.5 to 1.0 V) to identify the most efficient redox probe. The CV curve was then analyzed by referring to the Randles–Ševčík equation (Eq. (2))^127^, to determine the nature of the electrochemical interaction occurring at the electrode surface and assess the electroactive surface area of the modified electrodes.

$${I}_{p}=\left(2.69\times {10}^{5}\right){{n}_{{{{\rm{electron}}}}}}^{\frac{3}{2}}A{D}^{\,\frac{1}{2}}C{V}^{\frac{1}{2}}\,{{{\rm{at}}}}\,25\,^\circ {{{\rm{C}}}}$$

2 $$A=\frac{{I}_{p}}{\left(2.69\,\times \,{10}^{5}\right)\,{{n}_{{{{\rm{electron}}}}}}^{\frac{3}{2}}{D}^{\,\frac{1}{2}}C{V}^{\frac{1}{2}}}$$

The Randles–Ševčík equation (Eq. (2)) relates the peak current (*I*_p_) to several key parameters: *n*_electron_ represents the number of electrons transferred in the redox reaction, *A* denotes the electroactive surface area of the electrode, *D* is the diffusion coefficient of the redox species (for K_3_[Fe(CN)]_6_, *D* = 7.6 × 10^–6^ cm^2^ s^–1^), *C* corresponds to the bulk concentration of the electrolyte, and *V* signifies the applied scan rate.

After determining the optimal electrolyte configuration, all modified Co/Mn ZIF electrodes were evaluated using 70 µL of the optimum concentration (5 mM K_3_[Fe(CN)_6_] with 0.1 M KCl in 0.01 M PBS) through CV and EIS measurements. For CV, the scan rate was set ranging from 2 to 1000 mV s^–1^ throughout a potential range of –0.5 to 1.0 V to evaluate the nature reaction process through the linearity analysis on *I*_p_ vs. *V*^1/2^ and the log–log plot of *I*_pa_ vs. *V*. The Randles–Ševčík equation (Eq. (2)) was used to determine the electroactive surface area of Co/Mn ZIFs from the optimum CV curve. Furthermore, a selected frequency range between 10^–2^ and 10^6 ^Hz was applied for EIS characterization, with *R*_ct_ analysis performed to evaluate the EIS Nyquist plots based on charge transfer resistance.

### Hydrostability test of Co/Mn ZIF 2:1

To evaluate the hydrostability of the material, 7 mg of Co/Mn ZIF 2:1 powder was incubated in a glass containing 10 mL of distilled water (aqua bidest) for seven days. The samples were then centrifuged, washed with methanol, and dried at 60 °C for 48 h. The collected sample was evaluated through XRD by determining the presence of the related crystal plane, *d* spacing value (Eq. (1), and the most prominent peak area (011) plane by calculating the total intensity under the peak and above the baseline^103^.

### Anti-O antibody immobilization on SPCE/Co/Mn ZIF 2:1

The anti-O157 antibody was appointed in this study to sense *E. coli* O157:H7, a well known Shiga–toxin-producing strain associated with foodborne and contaminated water illnesses selectively. Using the physical adsorption technique, 10 µL anti-O antibody (2 µg mL^–1^) was deposited onto the optimized SPCE/Co/Mn ZIF 2:1 electrode, creating an SPCE/Co/Mn ZIF 2:1/anti-O electrode, which was then stored at a temperature of 4 °C for 16 h. After incubation, the modified electrode was rinsed using PBS and dried afterward. To prevent non-specific binding sites, 5 µL BSA was applied to the electrode. Incubation at 4 °C for 30 min forming SPCE/Co/Mn ZIF 2:1/anti-O/BSA electrode was followed by further rinsing in PBS. Antibody immobilization was comprehensively evaluated through multiple analytical techniques. Surface hydrophobicity was assessed via WCA measurement to evaluate changes in the interfacial properties upon antibody attachment. Chemical composition verification was performed using ATR-FTIR spectroscopy (Bruker Alpha, Germany), which confirmed the presence of a characteristic antibody functional group. An electrochemical evaluation was conducted through DPV measurements (PalmSens potentiostat, Netherlands) in an electrolyte solution containing 5 mM K_3_[Fe(CN)_6_] with 0.1 M KCl in 0.01 M PBS, employing a scan rate of 20 mV s^–1^ over a potential range of –0.5 to 1.0 V. DPV characterized antibody immobilization by measuring [Fe(CN)_6_]^3–/4–^ peak current attenuation, quantifying the blocking effect on electron transfer.

### *E. coli* detection using SPCE/Co/Mn ZIF 2:1/anti-O/BSA electrode

The modified SPCE/Co/Mn ZIF 2:1/anti-O/BSA electrode was hybridized with 10 µL of different concentrations of *E. coli* (10, 10^2^,10^4^, 10^6^, 10^8^, 10^10^ CFU mL^–1^) and then incubated at 4 °C for an hour. After incubation, PBS rinsing was applied to wash the electrode, followed by DPV measurements by dropping 70 µL of electrolyte (5 mM K_3_[Fe(CN)_6_] with 0.1 M KCl in 0.01 M PBS) across all three-electrode systems. DPV measurements were performed over a potential range of –0.5 to 1.0 V and a scan rate of 20 mV s^–1^. A calibration curve was obtained to provide the slope, which is essential for calculating the LoD:

3 $${{\rm{LoD}}}=\frac{3\it{S}_{{{{\rm{b}}}}}}{\it{m}}$$

where *S*_b_ represents the SD of the blank responses and *m* stands for the slope obtained from the calibration curve^128^.

### Analytical performance evaluation of SPCE/Co/Mn ZIF 2:1/anti-O/BSA electrode

The biosensor’s performance was thoroughly characterized using DPV to evaluate key analytical parameters (i.e., selectivity, stability, reproducibility, and recovery). In this evaluation, DPV measurements were performed over a potential range of –0.5 to 1.0 V and a scan rate of 20 mV s^–1^. Selectivity was assessed against non-target bacterial strains (i.e., a blank sample as the control test containing PBS 0.01 M, *Salmonella, Pseudomonas aeruginosa*, and *Staphylococcus aureus* at 10^6^ CFU mL^–1^ in 0.01 M PBS), as their frequent association with waterborne or clinical infections. For the long-term stability, it was evaluated by testing SPCE/Co/Mn ZIF 2:1/anti-O/BSA electrodes against 10^6^ CFU mL^–1^ of *E. coli* after storage at 4 °C for 1–7 weeks, with the signal retention calculated using the formula:

4 $$\% \,{{{\rm{stability}}}\; {{\rm{assay}}}}=\frac{{{{{I}}}}_{{{{\bf{0}}}}}-{{{{I}}}}_{{{{\bf{t}}}}}}{{{{{I}}}}_{{{{\bf{0}}}}}}\times {{{100}}} \%$$

where *I*_0_ denotes the initial current response, while *I*_t_ represents the current response at specific weekly intervals^129^. Furthermore, to validate the consistency of the biosensor fabrication process, reproducibility tests were performed using five independently prepared SPCE/Co/Mn ZIF 2:1/anti-O/BSA electrodes from the same production batch. For practical application validation, the electrodes were tested against *E. coli* at 10^6^ CFU mL^–1^. Recovery studies were performed by spiking tap water samples with known concentrations of *E. coli* (10^2^, 10^4^, 10^6^ CFU mL^–1^) to evaluate the biosensor accuracy in a complex matrix compared to the standard 0.01 M PBS solvent. A percentage recovery could be found by this formula^124,125^:

5 $$\% \,{{\rm{recovery}}}=\frac{{{\rm{concentration}}}\; {{\rm{found}}}\; {{\rm{in}}}\; {{\rm{spiked}}}\; {{\rm{sample}}}}{{{\rm{theoretical}}}\; {{\rm{concentration}}}}\times 100 \%$$

The DPV current response on the spiked sample represents the specific concentration found in the spiked sample, where the response obtained from 0.01 M PBS performs the theoretical value in the specific concentration, as expected that the biosensor exhibits an optimal and ideal environment.

### Ethics

All collaborators of this study have fulfilled the criteria for authorship required by Nature Portfolio journals and have been included as authors, as their participation was essential for the design and implementation of the study. Roles, responsibilities, and contributions were agreed among collaborators ahead of the research and manuscript writing. This research was not severely restricted or prohibited in the setting of the researchers, and did not result in stigmatization, incrimination, discrimination or personal risk to researchers. The references that are relevant and cited in this work not only come from our previous studies, but also include the reports from other research groups.

### Reporting Summary

Further information on research design is available in the Nature Portfolio Reporting Summary linked to this article.

## Supplementary information

Supplementary Information

Description of Additional Supplementary Files

Supplementary Data

Life Sciences Reporting Summary

## Data Availability

All data supporting the findings of this study are available within the article and Supplementary Information files. They are also available from the corresponding authors upon reasonable request.

## Code availability

The codes used in this study are available from the corresponding authors upon reasonable request.
