# Supplementary material for: High-performance electrochemical biosensor comprising Mn-ZIF-67 conjugated with anti-O antibody for Escherichia coli detection
Source: Commun Chem. 2025 Oct 1;8:290. doi: 10.1038/s42004-025-01703-y (PMC12488927; doi:10.1038/s42004-025-01703-y)
Supplement: Supplementary file 1 — Supplementary Information [file 42004_2025_1703_MOESM1_ESM.pdf]

# Supplementary Information

## High-performance electrochemical biosensor comprising Mn–ZIF–67 conjugated with anti–O antibody for Escherichia coli detection

**Atqiya Muslihati[1,2,3], Chandra Wulandari[1,2,3], Ni Luh Wulan Septiani[4], Gilang Gumilar[4], Agus Subagio[5], Ida Hamidah[6], Nugraha Nugraha[2,7], Erwin Peiner[8,*], Hutomo Suryo Wasisto[3,*], and Brian Yuliarto[2,7,*]**

[1] Doctoral Program of Engineering Physics, Faculty of Industrial Technology, Institut Teknologi Bandung, Jl. Ganesha No. 10, Bandung 41032, Indonesia

[2] Advanced Functional Material Laboratory, Faculty of Industrial Technology, Institut Teknologi Bandung, Jl. Ganesha No. 10, Bandung 41032, Indonesia

[3] PT Biostark Analitika Inovasi, Bandung 40375, Indonesia

[4] Research Center for Electronics, National Research and Innovation Agency (BRIN), Bandung 40135, Indonesia

[5] Department of Physics, Faculty of Science and Mathematics, Universitas Diponegoro Jl. Prof. Soedarto, Semarang 50275, Indonesia

[6] Department of Engineering and Vocational Education, Universitas Pendidikan Indonesia, Jl. Dr. Setiabudhi 207, Bandung 40154, Indonesia

[7] Research Center for Nanosciences and Nanotechnology (RCNN), Institut Teknologi Bandung, Jl. Ganesha No. 10, Bandung 41032, Indonesia

[8] Institute of Semiconductor Technology (IHT) and Laboratory for Emerging Nanometrology (LENA), Technische Universität Braunschweig, Hans–Sommer–Straße 66, Braunschweig 38106, Germany

[*] Corresponding authors. E–mails: e.peiner@tu–braunschweig.de (E.P.), h.wasisto@biostark–ai.com (H.S.W.), brian@itb.ac.id (B.Y.)

## Supplementary Note 1: Scanning electron microscopy (SEM)

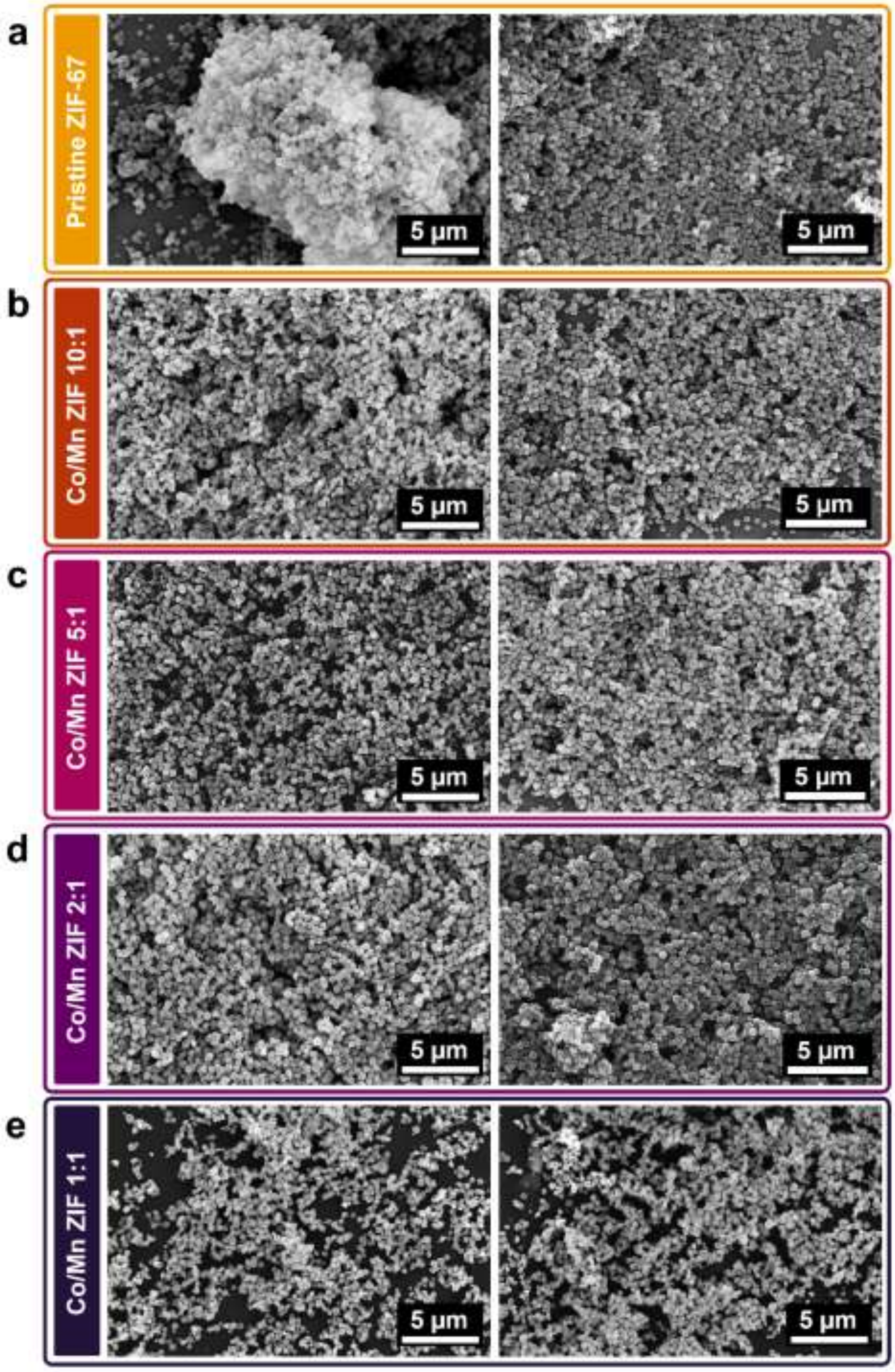


**Supplementary Figure 1. Scanning electron microscopy (SEM) images of Co/Mn ZIF samples with varied Co/Mn ratios.** SEM images with a magnification of 5000× on different spots were taken for **a** pristine ZIF–67, **b** Co/Mn ZIF 10:1, **c** Co/Mn ZIF 5:1, **d** Co/Mn ZIF 2:1, and **e** Co/Mn ZIF 1:1 sample. Here, consistent particle size distribution has been demonstrated across all samples, in which quantitative analysis is presented in the main article (see **Figure 2**).

## Supplementary Note 2: Cyclic voltammetry (CV)

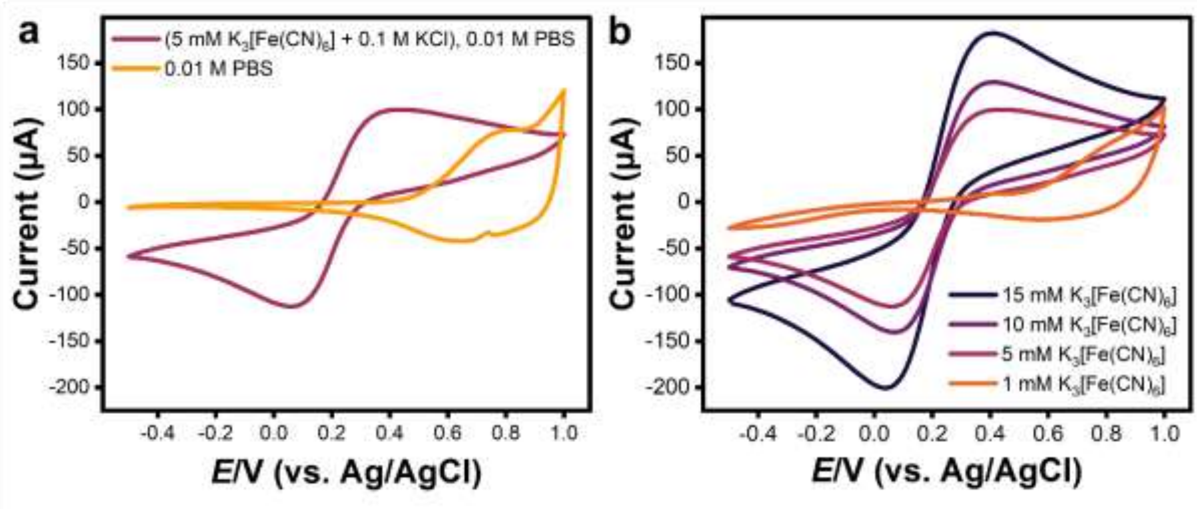


**Supplementary Figure 2. Cyclic voltammetry (CV) curves of screen–printed carbon electrode (SPCE/Co/Mn ZIF 2:1) electrode on different electrolytes and concentrations (scan rate of 20 mV $s^{-1}$, potential range of –0.5 to 1 V).** **a** CV curve of hexacyanoferrate (III)/(II) ($[Fe(CN)_6]^{3-/4-}$) redox reaction compared to that of 0.01 M phosphate buffer saline (PBS) electrolyte. **b** CV curve of $[Fe(CN)_6]^{3-/4-}$ redox reaction in different concentrations of potassium ferricyanide ($K_3[Fe(CN)_6]$) (1–15 mM) with 0.1 M potassium chloride (KCl) in 0.01 M PBS.

**Supplementary Table 1**. **Anodic peak current ($I_{pa}$) of $[Fe(CN)_6]^{3-/4-}$ redox reaction measured on various modified screen–printed carbon electrodes (SPCEs).** The SPCE/Co/Mn ZIF 2:1 electrode demonstrates enhanced electron transfer with the highest ($I_{pa}$ = 124 µA) compared to: (i) control electrodes (bare SPCE, SPCE/ZIF–67), (ii) other SPCE/Co/Mn ZIF ratio (10:1, 5:1, 1:1), and (iii) reference bimetallic MOFs ($Fe^{III}$–HMOF–5, NiCo–MOF, CoNi–MOF).

| Material | $I_{pa}$ (µA) |
|---|---|
| Bare SPCE | 44.30 |
| SPCE/ZIF–67 | 13.60 |
| SPCE/Co/Mn ZIF 10:1 | 17.80 |
| SPCE/Co/Mn ZIF 5:1 | 53.90 |
| SPCE/Co/Mn ZIF 2:1 | 124 |
| SPCE/Co/Mn ZIF 1:1 | 30.20 |
| $Fe^{III}$–HMOF–5 | ±40[1] |
| NiCo–MOF | ±80[2] |
| CoNi–MOF | ±115[3] |

**Supplementary Table 2. Measured peak currents ($I_p$) and potentials ($E_p$) from low to high scan rates CV.** The scan rates range from 2 to 1000 mV $s^{-1}$.

| Scan rate ($V$) (mV $s^{-1}$) | $I_{pa}$ (μA) | $I_{pc}$ (μA) | $I_{pa}/I_{pc}$ | $E_{pa}$ (V) | $E_{pc}$ (V) | $\Delta E_p$ (V) | $V^{1/2}$ | log ($V$) | log ($I_{pa}$) |
|---|---|---|---|---|---|---|---|---|---|
| 2 | 40.64 | –39.27 | –1.03 | 0.30 | 0.12 | 0.18 | 1 | 0.30 | 1.61 |
| 4 | 56.16 | –52.08 | –1.08 | 0.31 | 0.12 | 0.19 | 2 | 0.60 | 1.75 |
| 6 | 66.53 | –63.01 | –1.06 | 0.32 | 0.12 | 0.20 | 3 | 0.78 | 1.82 |
| 8 | 75.52 | –73.42 | –1.03 | 0.33 | 0.12 | 0.21 | 4 | 0.90 | 1.88 |
| 10 | 83.39 | –81.21 | –1.03 | 0.33 | 0.12 | 0.21 | 5 | 1.00 | 1.92 |
| 12 | 89.82 | –88.40 | –1.02 | 0.33 | 0.11 | 0.22 | 6 | 1.08 | 1.95 |
| 14 | 95.35 | –93.98 | –1.01 | 0.34 | 0.11 | 0.23 | 7 | 1.15 | 1.98 |
| 16 | 100.90 | –99.31 | –1.02 | 0.34 | 0.11 | 0.23 | 8 | 1.20 | 2.00 |
| 18 | 105.40 | –104.60 | –1.01 | 0.34 | 0.11 | 0.23 | 9 | 1.26 | 2.02 |
| 20 | 110.10 | –109.20 | –1.01 | 0.34 | 0.11 | 0.23 | 10 | 1.30 | 2.04 |
| 30 | 124.10 | –124.30 | –1.00 | 0.34 | 0.11 | 0.23 | 15 | 1.48 | 2.09 |
| 40 | 135.90 | –136.00 | –1.00 | 0.35 | 0.11 | 0.24 | 20 | 1.60 | 2.13 |
| 50 | 144.20 | –145.00 | –0.99 | 0.35 | 0.11 | 0.24 | 25 | 1.70 | 2.16 |
| 60 | 151.60 | –154.50 | –0.98 | 0.35 | 0.10 | 0.25 | 30 | 1.78 | 2.18 |
| 70 | 157.30 | –162.20 | –0.97 | 0.35 | 0.10 | 0.25 | 35 | 1.85 | 2.20 |
| 80 | 164.40 | –170.20 | –0.97 | 0.36 | 0.10 | 0.26 | 40 | 1.90 | 2.22 |
| 90 | 169.60 | –176.20 | –0.96 | 0.36 | 0.10 | 0.26 | 45 | 1.95 | 2.23 |
| 100 | 174.50 | –185.20 | –0.94 | 0.36 | 0.10 | 0.26 | 50 | 2.00 | 2.24 |
| 200 | 212.70 | –242.70 | –0.88 | 0.37 | 0.09 | 0.28 | 100 | 2.30 | 2.33 |
| 300 | 243.30 | –291.50 | –0.83 | 0.39 | 0.08 | 0.31 | 150 | 2.48 | 2.39 |
| 400 | 268.00 | –330.00 | –0.81 | 0.41 | 0.06 | 0.35 | 200 | 2.60 | 2.43 |
| 500 | 286.30 | –366.90 | –0.78 | 0.43 | 0.06 | 0.37 | 250 | 2.70 | 2.46 |
| 600 | 308.30 | –397.30 | –0.78 | 0.45 | 0.05 | 0.40 | 300 | 2.78 | 2.49 |
| 700 | 329.80 | –427.50 | –0.77 | 0.47 | 0.04 | 0.43 | 350 | 2.85 | 2.52 |
| 800 | 347.30 | –452.40 | –0.77 | 0.49 | 0.03 | 0.46 | 400 | 2.90 | 2.54 |
| 900 | 367.60 | –476.40 | –0.77 | 0.51 | 0.03 | 0.48 | 450 | 2.95 | 2.57 |
| 1000 | 381.50 | –499.00 | –0.76 | 0.53 | 0.02 | 0.51 | 500 | 3.00 | 2.58 |

## Supplementary Note 3: Electrochemical impedance spectroscopy (EIS)

**Supplementary Table 3. Electrochemical impedance spectroscopy (EIS) parameters of SPCE/Co/Mn ZIF electrodes in 5 mM $[Fe(CN)_6]^{3-/4-}$ electrolyte with 0.1 M KCl in 0.01 M PBS.** Measured resistances include solution resistance ($R_1$), total resistance at low frequency ($R_2$), and charge transfer resistance ($R_{ct}$). The SPCE/Co/Mn ZIF 2:1 electrode demonstrates optimal charge transfer kinetics, with $R_{ct}$ = 322 Ω.

| Parameter | Bare SPCE | SPCE/ZIF–67 | SPCE/Co/Mn ZIF 10:1 | SPCE/Co/Mn ZIF 5:1 | SPCE/Co/Mn ZIF 2:1 | SPCE/Co/Mn ZIF 1:1 |
|---|---|---|---|---|---|---|
| $R_1$ (Ω) | 24.48 | 32.71 | 49 | 30.29 | 32 | 40 |
| $R_2$ (Ω) | 4491 | 1098 | 410 | 418.60 | 354 | 387 |
| $R_{ct}$ (Ω) | 4466.52 | 1065.29 | 361 | 388.31 | 322 | 347 |

## Supplementary Note 4: Material hydrostability

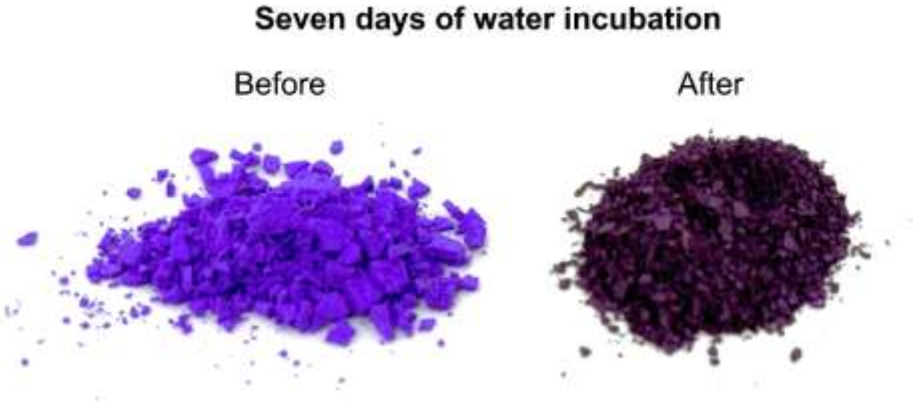


**Supplementary Figure 3. Pictorial representation of Co/Mn ZIF 2:1 before and after seven days of water incubation.** A gradual color change from bright to dark purple was observed, suggesting a little alteration in the coordination of the metal centers, potentially related to the ligand local symmetry [4,5].

## Supplementary Note 5: X–ray diffraction (XRD) analysis

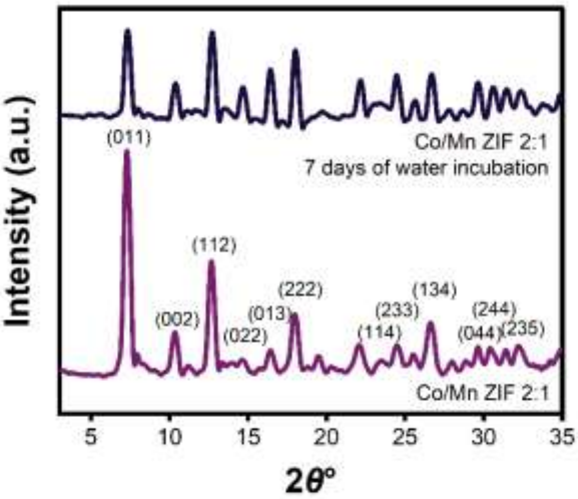


**Supplementary Figure 4. X–ray diffraction (XRD) patterns of Co/Mn ZIF 2:1 before and after seven days of water incubation.** The original material Co/Mn ZIF 2:1 was incubated for seven days in water to evaluate the hydrostability. After seven days of water incubation, all determined crystal planes of (011), (002), (112), (022), (013), (222), (114), (233), (134), (044), (244), and (235) remain detectable, indicating that the overall phase was preserved with no new peak appearing.

**Supplementary Table 4**. **Structural parameter of Co/Mn ZIF 2:1 before and after seven days of water incubation.** Minor decreases in *d* spacing (from 12.07 Å to 12.04 Å) and peak area (from 1456.18 to 574.82) were observed based on the most prominent (011) plane from the XRD pattern, suggesting a minor structural change in the remaining crystalline material, together with a significant loss of overall crystallinity.

| Sample | ***d*** **spacing (011)** (Å) | **Peak area (011)** |
|---|---|---|
| Co/Mn ZIF 2:1 | 12.07 | 1456.18 |
| Co/Mn ZIF 2:1–After 7 days water incubation | 12.04 | 574.82 |

## Supplementary Note 6: Water contact angle (WCA)

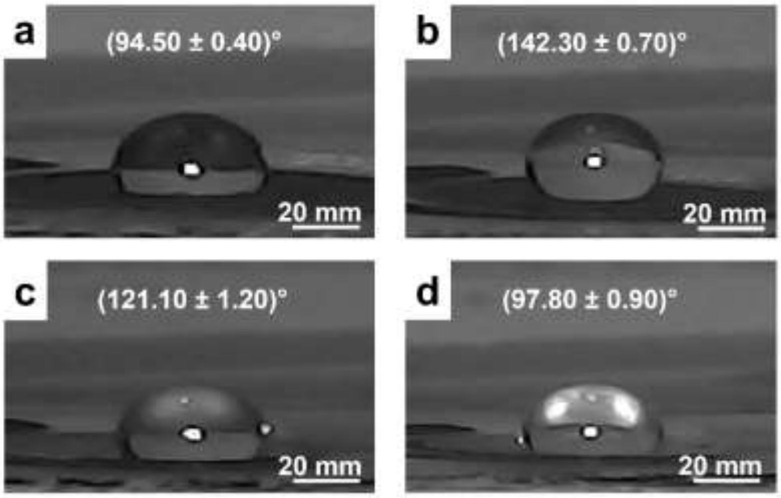


**Supplementary Figure 5. Surface wettability evolution of modified screen–printed carbon electrodes (SPCEs).** Water contact angle (WCA) measurements were conducted on the surfaces of **a** bare SPCE with WCA of (94.50 ± 0.40)°, **b** SPCE/Co/Mn ZIF 2:1 with WCA of (142.30 ± 0.70)° showing hydrophobic characteristic, **c** SPCE/Co/Mn ZIF 2:1/anti–O with WCA of (121.10 ± 1.20)° demonstrating hydrophilic conversion, and **d** SPCE/Co/Mn ZIF 2:1/anti–O/BSA with WCA of (97.80 ± 0.90)° exhibiting more hydrophilic property. WCA reduction after antibody immobilization creates a hydrated interface ideal for biosensing applications.

## Supplementary Note 7: Biosensor evaluation metrics

SPCE/Co/Mn ZIF 2:1/anti–O/BSA was directed to different pulse voltammetry (DPV) measurements on selectivity, stability, reproducibility, and recovery test. The measurement was performed using an electrolyte solution containing 5 mM $K_3[Fe(CN)_6]$ with 0.1 M KCl in 0.01 M PBS, at a potential range of –0.5 to 1.0 V and a scan rate of 20 mV $s^{-1}$.

**Supplementary Table 5. Selectivity current response of SPCE/Co/Mn ZIF 2:1/anti–O/BSA towards *E. coli* and other non–specific bacteria of *Salmonella, Pseudomonas,* and *Staphylococcus* ($10^6$ CFU $mL^{-1}$ in 0.01 M PBS) and blank (0.01 M PBS).** All non–target bacteria exhibit minimal deviation from the baseline current in the selectivity test, remaining below 35% of the *E. coli* current response.

| $n_{measurements}$ | Selectivity current response (%) | | | | |
|---|---|---|---|---|---|
| | *E. coli* | Blank | *Salmonella* | *Pseudomonas* | *Staphylococcus* |
| $n_1$ | 101.00 | 24.80 | 20.60 | 23.00 | 25.90 |
| $n_2$ | 98.60 | 28.30 | 26.50 | 30.40 | 28.10 |
| $n_3$ | 100.00 | 34.35 | 33.00 | 26.70 | 27.00 |
| **Mean ± SD** | (99.86 ± 1.20) | (29.15 ± 4.83) | (26.70 ± 6.20) | (26.70 ± 3.70) | (27.00 ± 1.10) |

**Supplementary Table 6. Stability of SPCE/Co/Mn ZIF 2:1/anti–O/BSA towards *E. coli* on different time periods of incubation.** The SPCE/Co/Mn ZIF 2:1/anti–O/BSA demonstrated good stability in *E. coli* detection ($10^6$ CFU $mL^{-1}$), maintaining more than 80% functionality up to 5 weeks despite slight performance degradation.

| $n_{measurements}$ | Stability (%) | | | | | | | |
|---|---|---|---|---|---|---|---|---|
| | Week 0 | Week 1 | Week 2 | Week 3 | Week 4 | Week 5 | Week 6 | Week 7 |
| $n_1$ | 101 | 93.20 | 97.70 | 90.70 | 91.20 | 85.40 | 75.70 | 65.40 |
| $n_2$ | 98.60 | 97.90 | 94.50 | 92.20 | 91.80 | 86 | 76.60 | 72.90 |
| $n_3$ | 100 | 94.50 | 92.20 | 94.90 | 93.50 | 91.30 | 78.70 | 77.10 |
| **Mean ± SD** | (99.86 ± 1.20) | (95.20 ± 2.42) | (94.80 ± 2.76) | (92.60 ± 2.12) | (92.16 ± 1.19) | (87.56 ± 3.24) | (77.00 ± 1.53) | (71.80 ± 5.92) |

**Supplementary Table 7. Reproducibility tests of the developed SPCE/Co/Mn ZIF 2:1/anti–O/BSA biosensors.** The current responses (μA) of SPCE/Co/Mn ZIF 2:1/anti–O/BSA towards $10^6$ CFU $mL^{-1}$ *E. coli* on five SPCE/Co/Mn ZIF 2:1/anti–O/BSA electrodes that were fabricated in the same batch. Excellent reproducibility was exhibited by the five fabricated electrodes, with a relative standard deviation (RSD) of 1.78%.

| $n_{measurements}$ | Current response (μA) | | | | |
|---|---|---|---|---|---|
| | Batch 1 | Batch 2 | Batch 3 | Batch 4 | Batch 5 |
| $n_1$ | 251 | 250 | 245 | 247 | 250 |
| $n_2$ | 261 | 254 | 253 | 255 | 254 |
| $n_3$ | 253 | 258 | 258 | 259 | 257 |
| **Mean ± SD** | (253.66 ± 4.53) | | | | |
| **Relative standard deviation (RSD) (%)** | 1.78 | | | | |

**Supplementary Table 8. Recovery of SPCE/Co/Mn ZIF 2:1/anti–O/BSA biosensors towards $10^6$ CFU mL$^{-1}$ *E. coli* spiked in tap water.** A satisfaction in percentage recovery was achieved with recovery values close to 100% from 90.80% to 114.56% with RSD < 7%.

| $n_{measurements}$ | Recovery (%) | | |
|---|---|---|---|
| | $10^2$ CFU mL$^{-1}$ | $10^4$ CFU mL$^{-1}$ | $10^8$ CFU mL$^{-1}$ |
| $n_1$ | 98.10 | 100 | 99.10 |
| $n_2$ | 92.80 | 94.10 | 103.70 |
| $n_3$ | 91.20 | 93.70 | 106.54 |
| $n_4$ | 92.60 | 92.90 | 113.73 |
| $n_5$ | 90.80 | 91.90 | 114.56 |
| **Mean ± SD** | (93.10 ± 2.61) | (94.52 ± 2.84) | (107.52 ± 5.90) |
| **RSD (%)** | 3.13 | 3.36 | 6.15 |

# References


1. Fang, X. & Duan, R. Highly Sensitive Capsaicin Electrochemical Sensor Based on Bimetallic Metal–Organic Framework Nanocage. *Front Chem* **10**, (2022).

2. Mouhamed, A. A., Nadim, A. H., Mahmoud, A. M., Mostafa, N. M. & Eltanany, B. M. Bimetallic MOF–based electrochemical sensor for determination of paracetamol in spiked human plasma. *BMC Chem* **18**, 148 (2024).

3. Devaraj, R., Loganathan, A. K. & Krishnamoorthy, L. Development of an aptasensor for highly sensitive detection of cardiac troponin I using cobalt–nickel metal–organic framework (CoNi–MOF). *Heliyon* **10**, e33238 (2024).

4. Gulbransen, E. A. & Andrew, K. F. The Kinetics of the Oxidation of Cobalt. *J Electrochem Soc* **98**, 241 (1951).

5. Butt, F. S. *et al.* Superhydrophobic ZIF–67 with exceptional hydrostability. *Mater Today Adv* **20**, 100448 (2023).
