# Supplementary material for: High-performance electrochemical biosensor comprising Mn-ZIF-67 conjugated with anti-O antibody for Escherichia coli detection
Source: Commun Chem. 2025 Oct 1;8:290. doi: 10.1038/s42004-025-01703-y (PMC12488927; doi:10.1038/s42004-025-01703-y)
Supplement: Supplementary file 2 — Description of Additional Supplementary Files [file 42004_2025_1703_MOESM2_ESM.pdf]

Description of Additional Supplementary Files

File name: Supplementary Data 1

Description: CIF 7222297
