# Supplementary material for: High-performance electrochemical biosensor comprising Mn-ZIF-67 conjugated with anti-O antibody for Escherichia coli detection
Source: Commun Chem. 2025 Oct 1;8:290. doi: 10.1038/s42004-025-01703-y (PMC12488927; doi:10.1038/s42004-025-01703-y)
Supplement: Supplementary file 4 — Life Sciences Reporting Summary [file 42004_2025_1703_MOESM4_ESM.pdf]

nature portfolio

Corresponding author(s): Erwin Peiner, Hutomo Suryo Wasisto, and Brian Yuliarto

Last updated by author(s): Aug 31, 2025

Reporting Summary

Nature Portfolio wishes to improve the reproducibility of the work that we publish. This form provides structure for consistency and transparency in reporting. For further information on Nature Portfolio policies, see our Editorial Policies and the Editorial Policy Checklist.

Statistics

For all statistical analyses, confirm that the following items are present in the figure legend, table legend, main text, or Methods section.

n/a	Confirmed	
☐	☒	The exact sample size (*n*) for each experimental group/condition, given as a discrete number and unit of measurement
☐	☒	A statement on whether measurements were taken from distinct samples or whether the same sample was measured repeatedly
☐	☒	The statistical test(s) used AND whether they are one- or two-sided<br>*Only common tests should be described solely by name; describe more complex techniques in the Methods section.*
☒	☐	A description of all covariates tested
☒	☐	A description of any assumptions or corrections, such as tests of normality and adjustment for multiple comparisons
☐	☒	A full description of the statistical parameters including central tendency (e.g. means) or other basic estimates (e.g. regression coefficient) AND variation (e.g. standard deviation) or associated estimates of uncertainty (e.g. confidence intervals)
☒	☐	For null hypothesis testing, the test statistic (e.g. *F*, *t*, *r*) with confidence intervals, effect sizes, degrees of freedom and *P* value noted<br>*Give P values as exact values whenever suitable.*
☒	☐	For Bayesian analysis, information on the choice of priors and Markov chain Monte Carlo settings
☒	☐	For hierarchical and complex designs, identification of the appropriate level for tests and full reporting of outcomes
☐	☒	Estimates of effect sizes (e.g. Cohen's *d*, Pearson's *r*), indicating how they were calculated

Our web collection on statistics for biologists contains articles on many of the points above.

Software and code

Policy information about availability of computer code

Data collection	Crystallographic data were obtained from the Crystallographic Open Database (COD, 7222297).
Data analysis	Statistical data analysis and figure preparation were conducted using OriginPro (v2023b, OriginLab Corporation).

For manuscripts utilizing custom algorithms or software that are central to the research but not yet described in published literature, software must be made available to editors and reviewers. We strongly encourage code deposition in a community repository (e.g. GitHub). See the Nature Portfolio guidelines for submitting code & software for further information.

Data

Policy information about availability of data

All manuscripts must include a data availability statement. This statement should provide the following information, where applicable:

- Accession codes, unique identifiers, or web links for publicly available datasets
- A description of any restrictions on data availability
- For clinical datasets or third party data, please ensure that the statement adheres to our policy

All data supporting the findings of this study are available within the article and Supplementary Information files. They are also available from the corresponding authors upon reasonable request.

Research involving human participants, their data, or biological material

Policy information about studies with human participants or human data. See also policy information about sex, gender (identity/presentation), and sexual orientation and race, ethnicity and racism.

Reporting on sex and gender	This information has not been collected.
Reporting on race, ethnicity, or other socially relevant groupings	*Please specify the socially constructed or socially relevant categorization variable(s) used in your manuscript and explain why they were used. Please note that such variables should not be used as proxies for other socially constructed/relevant variables (for example, race or ethnicity should not be used as a proxy for socioeconomic status).*<br>*Provide clear definitions of the relevant terms used, how they were provided (by the participants/respondents, the researchers, or third parties), and the method(s) used to classify people into the different categories (e.g. self-report, census or administrative data, social media data, etc.)*<br>*Please provide details about how you controlled for confounding variables in your analyses.*
Population characteristics	*Describe the covariate-relevant population characteristics of the human research participants (e.g. age, genotypic information, past and current diagnosis and treatment categories). If you filled out the behavioural & social sciences study design questions and have nothing to add here, write "See above."*
Recruitment	*Describe how participants were recruited. Outline any potential self-selection bias or other biases that may be present and how these are likely to impact results.*
Ethics oversight	*Identify the organization(s) that approved the study protocol.*

Note that full information on the approval of the study protocol must also be provided in the manuscript.

Field-specific reporting

Please select the one below that is the best fit for your research. If you are not sure, read the appropriate sections before making your selection.

☒ Life sciences ☐ Behavioural & social sciences ☐ Ecological, evolutionary & environmental sciences

For a reference copy of the document with all sections, see nature.com/documents/nr-reporting-summary-flat.pdf

Life sciences study design

All studies must disclose on these points even when the disclosure is negative.

Sample size	Sample size was determined based on preliminary trials showing consistent detection of E. coli using Co/Mn ZIF based-MOF-anti-O antibody conjugates throughout a minimum of three independent tests. No formal statistical method was used to predetermine the sample size, as this is a proof-of-concept study. There were enough repetitions (n = 3–5) to ensure reproducibility and evaluate performance consistency.
Data exclusions	The analysis did not exclude any data. The final results contained all of experimental data points that were collected. Exclusion criteria were not applicable to this study.
Replication	To ensure the reproducibility, each experiment was performed three times using independently prepared samples. Results showed consistent detection performance across all replicates.
Randomization	Randomization was not applicable as the study did not involve treatment groups, clinical subjects, or biological diversity in test populations. Under controlled laboratory condition, samples were processed consistently.
Blinding	Blinding was not relevant to this study, as the experimental procedures and analyses were conducted objectively using predetermined protocols, and detection was performed via measurable output (e.g., signal current) without subjective judgment.

Reporting for specific materials, systems and methods

We require information from authors about some types of materials, experimental systems and methods used in many studies. Here, indicate whether each material, system or method listed is relevant to your study. If you are not sure if a list item applies to your research, read the appropriate section before selecting a response.

Materials & experimental systems

n/a	Involved in the study
☐	☒ Antibodies
☒	☐ Eukaryotic cell lines
☒	☐ Palaeontology and archaeology
☒	☐ Animals and other organisms
☒	☐ Clinical data
☒	☐ Dual use research of concern
☒	☐ Plants

Methods

n/a	Involved in the study
☒	☐ ChIP-seq
☒	☐ Flow cytometry
☒	☐ MRI-based neuroimaging

Antibodies

Antibodies used

Anti-O157 E. coli monoclonal antibody (MyBioSource MBS 530858).

Validation

An IgG2b isotype derived from a mouse host, demonstrates specificity for Escherichia coli O157. It is supplied as a liquid in 10 mM PBS, pH 7.2, containing 0.1% sodium azide as a preservative, and is greater than 90% pure, with a concentration of 2.3 mg/mL (lot specific). The antibody recognizes the 'O' antigen of E. coli serotype O157. E. coli is a Gram-negative rod-shaped bacterium typically found in the lower intestines of warm-blooded organisms (endotherms). While most strains are harmless and part of the normal gut flora—contributing to host health by producing vitamin K2 and preventing colonization by pathogenic bacteria—some serotypes, such as O157:H7, are pathogenic and can cause serious foodborne illness in humans and are linked to product recalls. The immunogen used to produce this antibody was E. coli serotype O157. For storage, the antibody should be kept at 4°C for short-term or –20°C for long-term preservation.

Plants

Seed stocks

This information has not been collected.

Novel plant genotypes

Describe the methods by which all novel plant genotypes were produced. This includes those generated by transgenic approaches, gene editing, chemical/radiation-based mutagenesis and hybridization. For transgenic lines, describe the transformation method, the number of independent lines analyzed and the generation upon which experiments were performed. For gene-edited lines, describe the editor used, the endogenous sequence targeted for editing, the targeting guide RNA sequence (if applicable) and how the editor was applied.

Authentication

Describe any authentication procedures for each seed stock used or novel genotype generated. Describe any experiments used to assess the effect of a mutation and, where applicable, how potential secondary effects (e.g. second site T-DNA insertions, mosiacism, off-target gene editing) were examined.
